# L3MBTL2-mediated CGA transcriptional suppression promotes pancreatic cancer progression through modulating autophagy

**DOI:** 10.1016/j.isci.2022.104249

**Published:** 2022-04-13

**Authors:** Hua Huang, Ruining Pan, Yue Zhao, Huan Li, Huiyu Zhu, Sijia Wang, Aamir Ali Khan, Juan Wang, Xinhui Liu

**Affiliations:** 1 Center of Excellence for Environmental Safety and Biological Effects, Beijing International Science and Technology Cooperation Base for Antiviral Drugs, Faculty of Environment and Life, Beijing University of Technology, Beijing 100124, China; 2 Intensive Care Unit, Beijing Tsinghua Changgung Hospital, Beijing 102218, China; 3 School of Pharmacy, Henan University of Chinese Medicine, Zhengzhou 450046, China

**Keywords:** Biological sciences, Cell biology, Cancer

## Abstract

L3MBTL2 is a crucial component of ncPRC1.6 and has been implicated in transcriptional repression and chromatin compaction. However, the repression mechanism of L3MBTL2 and its biological functions are largely undefined. Here, we found that L3MBTL2 plays a distinct oncogenic role in tumor development. We demonstrated that L3MBTL2 repressed downstream CGA through an H2AK119ub1-dependent mechanism. Importantly, the binding of the MGA/MAX heterodimer to the E-box on the CGA promoter enhanced the specific selective repression of CGA by L3MBTL2. CGA encodes the alpha subunit of glycoprotein hormones; however, we showed that CGA plays an individual tumor suppressor role in PDAC. Moreover, CGA-transcript1 (T1) was identified as the major transcript, and the tumor suppression function of CGA-T1 depends on its own glycosylation. Furthermore, glycosylated CGA-T1 inhibited PDAC, partly by repression of autophagy through multiple pathways, including PI3K/Akt/mTOR and TP53INP2 pathways. These findings reveal the important roles of L3MBTL2 and CGA in tumor development.

## Highlights

• L3MBTL2 plays a distinct oncogenic role in tumor development
• L3MBTL2 represses CGA transcription mainly by mediating ubiquitination of H2A
• CGA plays an individual tumor suppressor role in pancreatic cancer
• Glycosylated CGA inhibited PDAC partly through repression of autophagy

## Graphical abstract

**Figure:**
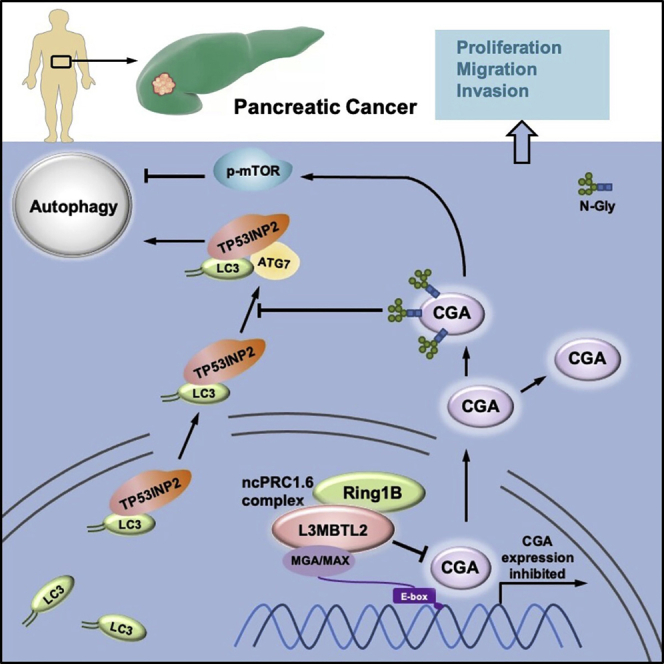

## Introduction

Pancreatic ductal adenocarcinoma (PDAC), among all other malignancies, is one of the primary causes of cancer-related death globally because of its characteristically deadly aftermath (Gomez-Rubio et al., 2017). The frequency of PDAC is substantially increasing worldwide, and it accounts for the worst prognosis among all digestive cancers because of its masked anatomical position, lack of effective treatment, and late clinical diagnosis. The average survival of a patient with PDAC is 2–8 months, and they only have a 6% chance of survival five years after diagnosis (Jemal et al., 2011). A deep understanding is a prerequisite to unveil the molecular mechanism, protein interactions, and genomic mutability to grasp how these factors are involved in the development and progression of pancreatic cancer, and this might be helpful for therapeutic developments.

Lethal-3-malignant brain tumor-like 2 (L3MBTL2) is an essential member of the Polycomb group proteins (PcG), and it belongs to the family of Malignant brain tumor (MBT) proteins that are associated with modification of the chromatin structure by binding to histones, which results in compaction of the chromatin (Bonasio et al., 2010; Qin et al., 2012; Trojer et al., 2007; Ogawa et al., 2002). L3MBTL2 possesses a zinc finger domain at the N-terminal end and four MBT domains, which facilitate the recognition of methylated histones (Guo et al., 2009). Notably, in contrast to L3MBTL1 that compacts nucleosomes only in the presence of methyl marks, L3MBTL2 is involved in chromatin compaction independent of histone modification (Frankenberg et al., 2007; Trojer et al., 2007, 2011). In addition, L3MBTL2 functions as a transcription suppressor because it is an important component of the Polycomb suppression Complex-1 family (PRC1), which is one of the two major PcG complexes (the other is PRC2) (Qin et al., 2012; Montellier et al., 2013; Yoo et al., 2010; Trojer et al., 2011). PRC1 and PRC2 catalyze two repressive histone modifications: monoubiquitination of histone H2A at lysine-119 (H2AK119ub1) and trimethylation of histone H3K27, respectively (Tavares et al., 2012; Muller and Verrijzer, 2009). The combined activity of PRC1 and PRC2 is considered to be critical for normal Polycomb-mediated transcriptional repression. In mammals, six independent groups of PRC1 complexes (PRC1.1-PRC1.6) have been identified based on the availability of the PCGF subunit, and L3MBTL2 is a component of the noncanonical PRC1.6 (ncPRC1.6) complex (Gao et al., 2012). Although it remains elusive how L3MBTL2 transcription represses its target genes, several recent studies have provided a mechanism by which L3MBTL2 interacts with the core constituents of PRC1.6 and facilitates recruitment of the PRC1.6 complex to the promoters of specific target genes (Huang et al., 2018b; Stielow et al., 2018).

The revealed biological functions of L3MBTL2 are still limited. L3MBTL2 has been shown to have an essential role in early embryonic development and pluripotent stem cells (Qin et al., 2012; Eid and Torres-Padilla, 2016). L3MBTL2 is highly expressed in testicular tissues and is involved in chromatin remodeling throughout meiosis and spermatogenesis (Meng et al., 2019). L3MBTL2 inhibits the DNA damage-p53-apoptosis pathway and therefore has been reported to play a protective role in kidney injury and silica nanoparticle-induced spermatogenesis disorders (Huang et al., 2018a; Liu et al., 2020; Nowsheen et al., 2018). However, the other biological functions of L3MBTL2 are largely undefined, and there is no direct evidence that L3MBTL2 plays a role in cancer (Khan et al., 2015).

Glycoprotein hormone alpha subunit (CGA) encodes the alpha subunit of the glycoprotein hormone family, which includes human chorionic gonadotropin (hCG), luteinizing hormone (LH), follicle-stimulating hormone (FSH), and thyroid-stimulating hormone (TSH) (Chin et al., 1981). These four glycoprotein hormones are composed of two noncovalently bound subunits labeled α and β. In contrast to the shared same α subunit, the β-subunit confers both receptor and biological specificity that are distinct for each glycoprotein hormone (Pierce and Parsons, 1981). Therefore, the majority of studies concerning their role and mechanism in cancer have focused on the β-subunits, such as β-hCG, which was shown to promote metastasis through the ERK/MMP2 pathway in ovarian cancer (Wu et al., 2019). Aberrant expression of CGA has been detected in gastric cancer, ER-positive breast cancer, and prostate cancer (Li et al., 2014; Bieche et al., 2001, 2002). However, the reasons for the aberrant CGA expression and whether CGA has an individual role in cancer are still elusive.

Autophagy is an evolutionarily conserved catabolic process that targets cellular organelles and macromolecules to lysosomal compartment for degradation, which plays a major role in cellular homeostasis and participates in a variety of biological activities (Kundu and Thompson, 2008). Dysfunctional autophagy is implicated in various forms of human disease. In cancer, the role of autophagy seems to be complex and biphasic, i.e., either tumor-suppressive or tumor-promoting role depending on tumor type or contexts (White and DiPaola, 2009). Accumulating evidence demonstrates that autophagy activation is prevalent in PDAC and meditated pancreatic cancer development (Tsang et al., 2020; Zhou et al., 2020). However, different from other cancers, pancreatic cancers have a distinct dependence on autophagy. A study by Yang et al. demonstrated that autophagy is required for tumorigenic growth of pancreatic cancers *de novo* and the continued malignant growth during the late stages of PDAC transformation (Yang et al., 2011). Therefore, the exploration of genes regulating autophagy in PDAC may provide new strategies for treatment.

In this study, we examined the contribution of L3MBTL2 to tumor progression. We demonstrated that L3MBTL2 mediates the repression of CGA by interacting with ncPRC1.6 complexes and regulating histone modifications at the CGA promoter. Importantly, the binding of the MGA/MAX heterodimer to the E-box on the CGA promoter enhanced the specific selective repression of CGA by L3MBTL2. We further demonstrated that CGA plays an individual tumor suppressor role in PDAC, which is independent of glycoprotein hormones. We also investigated the underlying mechanism of the individual tumor suppressor role of CGA in PDAC and indicated that the function of CGA depends on its alternative pre-mRNA splicing and protein glycosylation. Collectively, we proposed that L3MBTL2-mediated CGA repression facilitates tumorigenesis and it represents a potential therapeutic strategy.

## Results

### L3MBTL2 promotes tumor growth and metastasis in pancreatic cancer

**Figure 1:**
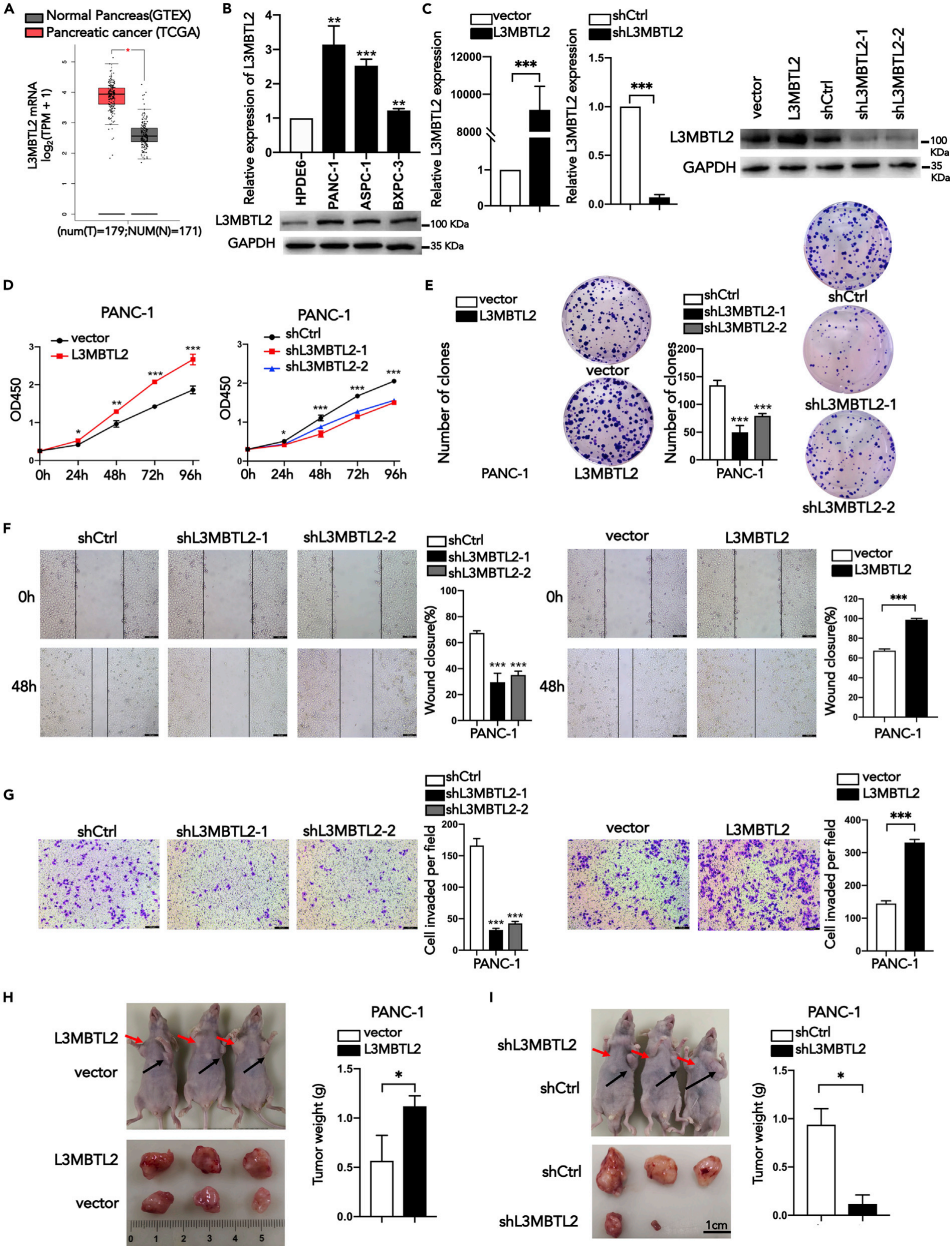
L3MBTL2 is required for the tumorigenesis and metastasis of PDAC cells (A) Box plot showing the relative mRNA levels of L3MBTL2 in pancreatic cancer tissues (n = 179) and nontumor tissues (n = 171) according to the TCGA and GTEX database. (B) The relative mRNA and protein levels of L3MBTL2 in pancreatic cancer cells and pancreatic ductal epithelial cells. (C) The overexpression and knockdown of L3MBTL2 in PANC-1 cells were confirmed by qPCR and western blotting. (D) Proliferation of PANC-1 cells expressing exogenous L3MBTL2, vector, shL3MBTL2(1,2), and shCtrl. (E) Representative images of the clone forming an assay of the indicated cells. (F and G), wound healing assays (F), Transwell assays (G) of PANC-1 cells expressing exogenous L3MBTL2, vector, shL3MBTL2(1,2), and shCtrl. Scale bars, 100 μm (H and I), the volumes and weight of subcutaneous tumors from the indicated groups. Data are presented as means ± SD (n = 3). ∗p < 0.05, ∗∗p < 0.01, ∗∗∗p < 0.001, t-tests.

The role of L3MBTL2 in cancer is still unclear. We first investigated the role of L3MBTL2 in PDAC, because the expression level of L3MBTL2 shows a greater discrepancy between tumor and adjacent tissues in PDAC than in other cancers (Figure S1). According to the TCGA and GTEx database, L3MBTL2 expression was preferentially higher in pancreatic adenocarcinoma (PAAD) tissues (n = 179) than in nontumor tissues (n = 171) (Figure 1A). To further verify the L3MBTL2 expression level in PDAC, we selected three pancreatic cancer cell lines (ASPC-1, BXPC-3, and PANC-1) and one pancreatic ductal epithelial cell line (HPDE6-C7). Quantitative PCR and western-blot detected increased L3MBTL2 mRNA and protein in PDAC cells compared to HPDE6-C7 cells, shedding light on the oncogenic role of L3MBTL2 in PDAC (Figure 1B.).

To comprehensively investigate the function of L3MBTL2 in PDAC, we established L3MBTL2 overexpression and knockdown stably transfected PANC-1 and ASPC-1 cell lines. Transfection was confirmed by analyzing the mRNA and protein levels of L3MBTL2 (Figure 1C). Upregulation of L3MBTL2 significantly promoted the proliferation, colony formation, cell migration and invasion capacity, and vice versa for its knockdown (Figures 1D–1G and S2A–S2C). In addition, we also established a subcutaneous xenograft mouse model to evaluate the role of L3MBTL2 in tumorigenesis *in vivo* (n = 3/group). The tumor weight and volume of the L3MBTL2 overexpression group were significantly increased, whereas L3MBTL2 knockdown had the opposite effect (Figures 1H and 1I). Collectively, these results demonstrated that L3MBTL2 promotes cell proliferation, migration, invasion, and tumorigenesis in both PANC-1 and ASPC-1 cells.

### L3MBTL2 transcriptional suppresses the expression of CGA

**Figure 2:**
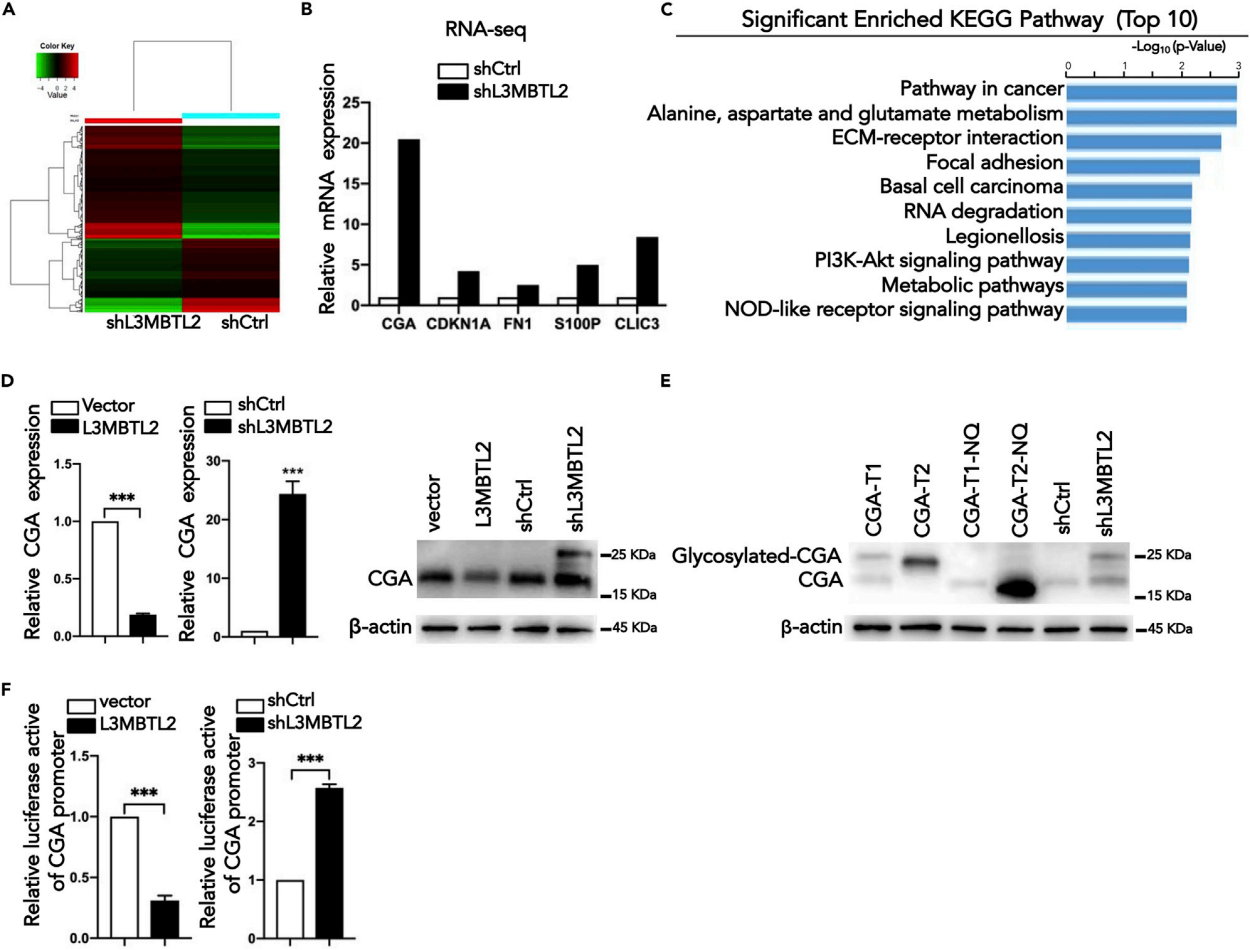
CGA is a downstream target of L3MBTL2 (A) RNA-seq were used to identify differentially expressed genes in L3MBTL2 stably knockdown cells when compared with their corresponding controls (fold change >2 or 2.0, p < 0.05). (B) The relative mRNA levels of CGA, CDKN1A, FN1, S100P, and CLIC3 in RNA-seq of PANC-1 cells after L3MBTL2 knockdown. (C) KEGG pathway enrichment analysis of DEGs was performed to identify functionally related gene pathways. The top 10 enriched signaling pathways are shown and are ranked on the basis of log10(P-value). (D) qPCR and western blotting were performed to determine the mRNA and protein levels of CGA in shL3MBTL2 and shCtrl cells. (E) Western blotting analysis of CGA in PANC-1 cells expressing exogenous CGA-T1, CGA-T1-NQ, CGA-T2, CGA-T2-NQ, shCtrl, and shL3MBTL2. (F) A luciferase reporter assay evaluated the effect of L3MBTL2 overexpression and knockdown on the transcriptional activity of CGA in PANC-1 cells. Firefly luciferase activity was normalized to Renilla luciferase activity and expressed as the mean ± SD. Data are presented as means ± SD (n = 3). ∗p < 0.05, ∗∗p < 0.01, ∗∗∗p < 0.001, t-tests.

L3MBTL2 has been suggested to function as a transcriptional repressor. We thus hypothesized that the oncogenic function of L3MBTL2 in PDAC may be closely related to its suppressive effect on key downstream target genes. To explore its downstream pathway, we performed RNA sequencing (RNA-seq) using PANC-1-shL3MBTL2 and the corresponding control cells (Figure 2A). We identified 650 differentially expressed genes (DEGs) in shL3MBTL2 cells relative to the control group (fold change≥2, p < 0.05). Many of these DEGs, including CGA, CDKN1A, FN1, CLIC3, and S100P, have been reported to play essential roles in cancer progression (Figure 2B). Subsequently, KEGG pathway enrichment analysis revealed that the Pathway in cancer was the top most enriched signaling pathway, and there were also other pathways closely related to tumor development, such as the PI3K-Akt signaling pathway, among the top 10 most enriched pathways (Figure 2C). This suggests that L3MBTL2 plays a critical role in cancer, and the abnormal expression of L3MBTL2 may be involved in multiple mechanisms of tumor development. Therefore, we assumed that the target gene of L3MBTL2 might be a tumor suppressor gene with a certain role in cancer. However, we found that among the DEGs, one of the genes most strongly upregulated in response to L3MBTL2 knockdown was a hormone-related gene (CGA), whose role in cancer is still unclear. This aroused our great interest. Subsequently, we confirmed that L3MBTL2 remarkably negatively regulated CGA expression at the mRNA and protein levels (Figure 2D). Notably, two CGA bands appeared in shL3MBTL2 cells, whereas there is only single band in other cells (Figure 2D). According to the NCBI database, CGA has two transcripts. Transcript 1 is a full-length transcript of 147 amino acids, and transcript 2 lacks 32 amino acids (30E∼61D). Besides, CGA encodes the alpha subunit of glycoprotein hormones, which is a glycosylated protein containing 92 amino acids with two N-glycosylation sites (107N and 133N). We hypothesized that L3MBTL2 may also inhibit the glycosylation of CGA. To test this, we established four stable overexpressed cell lines that overexpressed CGA transcript 1(CGA-T1), CGA transcript 2 (CGA-T2), CGA-Transcript1-N107Q-N133Q (CGA-T1-NQ), and CGA-Transcript2-N76Q-N102Q (CGA-T2-NQ), respectively (Figure 2E). CGA-T1-NQ and CGA-T2-NQ are mutations at two same N glycosylation sites. We found that CGA-T1 is the primary transcript in PANC-1 cells according to the protein weight (Figure 2E). Furthermore, the shL3MBTL2 promoted the expression of total CGA while also possibly promoting the glycosylation of CGA through other mechanisms. This explains why two CGA bands appeared in shL3MBTL2 cells. In addition, to evaluate the transcriptional regulatory role of L3MBTL2 on CGA, a luciferase promoter-reporter assay was performed. L3MBTL2 overexpression significantly decreased the activity of the CGA promoter, whereas L3MBTL2 knockdown had the opposite effect (Figure 2F). These results demonstrate that CGA is a direct target of L3MBTL2, which transcriptionally suppresses the CGA in pancreatic cancer.

### L3MBTL2 represses CGA by mediating ubiquitination of H2A

**Figure 3:**
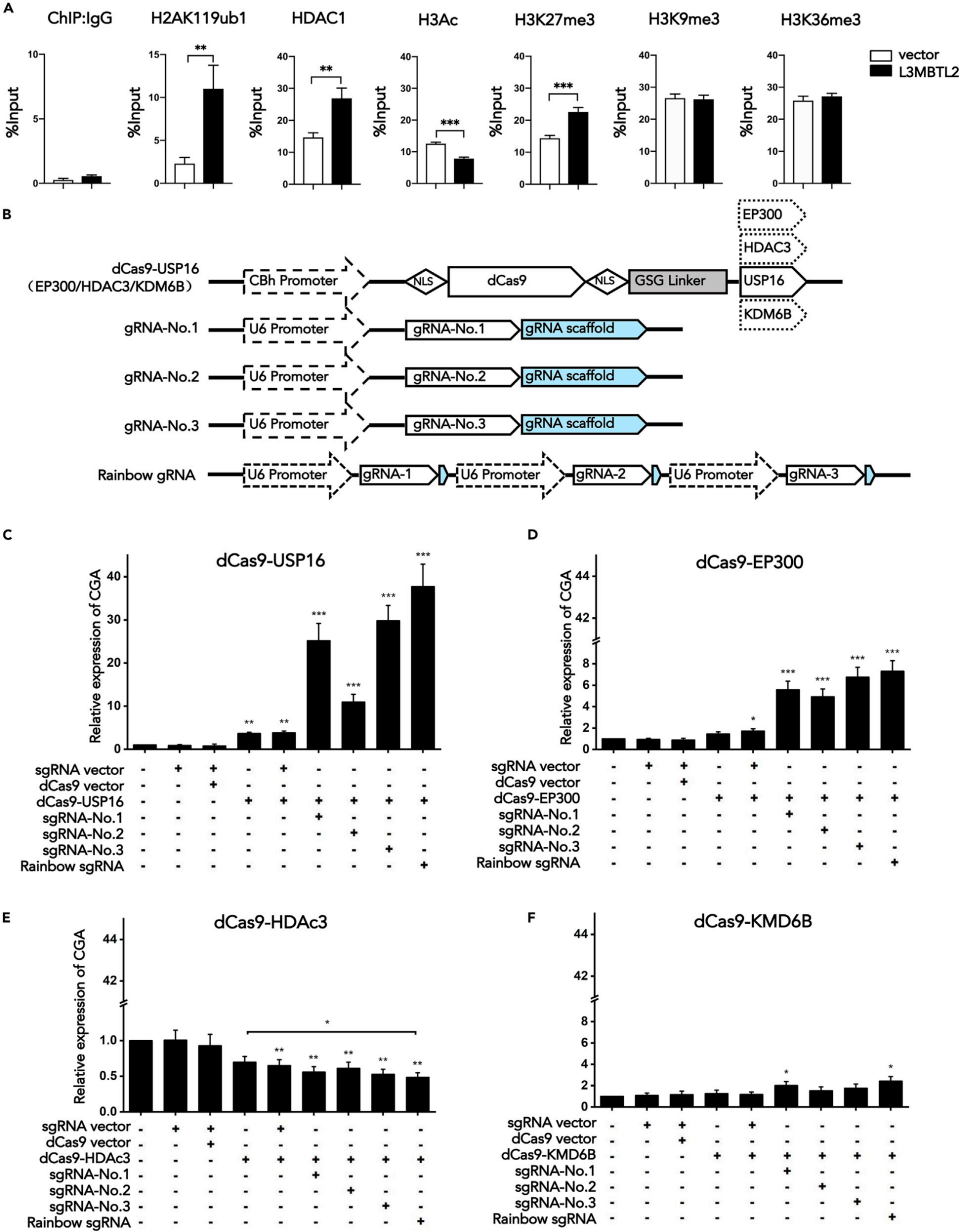
L3MBTL2 represses CGA by mediating histone modifications (A) Overexpression of L3MBTL2 correlates with increased H2AK119ub1, HDAC1, and H3K27me3 at the CGA promoter but does not affect H3K9me3 and H3K36me3. ChIP of IgG control antiserum, HDAC1, H3Ac, H3K27me3, H3K9me3, and H3K36me3 (left to right) followed by qPCR analysis of CGA in PANC-1 cells. (B) Schematic of dCas9 fusion constructs, dCas9-USP16, dCas9-EP300, dCas9-HDAC3, dCas9-KDM6B, and different gRNA constructs. (C, D, and F), Relative mRNA levels of CGA in L3MBTL2-overexpressing PANC-1 cells co-transfected with either dCas9 fusion proteins (dCas9-USP16 (C), dCas9-EP300 (D), and dCas9-KDM6B (F)) or dCas9 empty vector and the indicated gRNAs. (E) Relative mRNA levels of CGA in L3MBTL2 knockdown PANC-1 cells co-transfected with either dCas9-HDAC3 or dCas9 empty vector and the indicated gRNAs. Data are presented as means ± SD (n = 3). ∗p < 0.05, ∗∗p < 0.01, ∗∗∗p < 0.001, t-tests.

L3MBTL2 functions as a transcriptional repressor as L3MBTL2 has been reported to interact with the core components of ncPRC1.6 and contribute to the recruitment of this complex to the promoter of target genes. To investigate the local impact of L3MBTL2 on chromatin modifications at the CGA promoter, we performed ChIP analysis. All PRC1 complexes, including PRC1.6, contained RING1B, which catalyzes H2AK119ub1 and thereby contributes to chromatin compaction and transcriptional silencing. Therefore, we investigated whether L3MBTL2 represses CGA by mediating H2AK119ub1. As expected, ChIP-qPCR analysis revealed that H2AK119ub1 at the CGA promoter was markedly increased after overexpression of L3MBTL2, revealing a role for L3MBTL2 in CGA repression by mediating H2AK119ub1 deposition (Figure 3A). In addition, histone acetylation has been suggested to be involved in transcriptional gene regulation, and HDACs have been identified to interact with L3MBTL2 (Qin et al., 2012; Yoo et al., 2010). We therefore investigated whether L3MBTL2-mediated CGA inhibition was related to histone acetylation. The expression of HDAC1, a histone deacetylase, was increased after L3MBTL2 overexpression, indicating that histone acetylation (H3Ac and H4Ac) at the CGA promoter was decreased. Indeed, we confirmed the decreased acetylation changes by detecting H3Ac on CGA promoter. Moreover, H3K27me3 was increased at the CGA promoter. The increase of H3K27me3 may be the result of an increase in H2AK119ub1, which has been indicated to recruit PRC2 and downstream H3K27me3 deposition to effectively initiate a Polycomb domain (Blackledge et al., 2014). Other histone modifications, such as H3K9me3 and H3K36me3, were virtually unchanged at the CGA promoter. Collectively, the upregulation of L3MBTL2 resulted in a significant increase in H2AK119ub1 on the CGA promoter, whereas the changes in histone acetylation and H3K27me3 were relatively minor.

To gain insight into the functional involvement of L3MBTL2-mediated H2AK119ub1, acetylation, and H3K27me3 deposition in CGA transcription, we further rescued these histone modifications upstream of the TSS of the CGA promoter and evaluated the effects of their alterations on the CGA transcription level. A CRISPR-Cas9-based fusion system was applied to modify histone modifications of the CGA promoter. USP16, a deubiquitinating enzyme, was fused with the dCas9 protein by a GSG flexible connection linker (dCas9-USP16), which decreased H2AK119ub1 at the target loci. In addition, 3 independent gRNAs were designed to lead dCas9-USP16 to upstream of the TATA box of the CGA promoter. Furthermore, we combined three gRNA transcription frames into a single vector, named Rainbow gRNA, to shield the problem of uneven efficiency in co-transfection (Figures 3B and S3). The specificity and effectiveness of gRNAs were verified by co-transfected dCas9-USP16 and gRNAs into 293T^RING1B−OE^ cells and analyzed the H2AK119ub1 levels at the CGA promoter by ChIP-qPCR (Figure S4).

L3MBTL2-overexpressing PANC-1 cells transfected with dCas9-USP16 alone or co-transfected with a scrambled gRNA barely affected L3MBTL2-mediated CGA repression. Notably, L3MBTL2-overexpressing cells co-transfected with the dCas9-USP16 fusion construct. and each of the 4 gRNAs failed to maintain transcriptional repression, indicating that L3MBTL2-mediated repression of CGA was significantly abolished by the loss of H2AK119ub1 deposition at the CGA promoter (Figure 3C). Subsequently, the fusion of dCas9 with histone demethylase KDM6B, histone deacetylase HDAC3, and acetyltransferase EP300 was constructed in a similar way to decrease H3K27me3 and to reduce and increase histone acetylation (H3Ac and H4Ac) at the CGA promoter, respectively. The results showed that the CGA repressed by L3MBTL2-OE (L3MBTL2-overexpressing cells) was partially rescued by the increase in histone acetylation at the CGA promoter (Figure 3D). In contrast, the reduced histone acetylation at the CGA promoter inhibited CGA transcription (Figure 3E). However, compared with the fold change of CGA expression affected by histone ubiquitination, the effect of histone acetylation on CGA was obviously weaker. In addition, decreased H3K27me3 at the CGA promoter induced by dCas9-KDM6B transfection of L3MBTL2-OE cells did not impact CGA transcription, suggesting that L3MBTL2-mediated CGA repression is H3K27me3 independent (Figure 3F). Collectively, L3MBTL2-mediated CGA repression was significantly abolished by a loss of H2AK119ub1 deposition at the CGA promoter but was partially rescued by increased histone acetylation independent of H3K27me3. Thus, we suggested that L3MBTL2 represses CGA mainly by regulating H2AK119ub1, whereas changes in acetylation (H3Ac and H4Ac) and H3K27me3 may contribute to optimal L3MBTL2-mediated repression of CGA.

### The E-boxes on the CGA promoter are essential for specific repression of CGA by L3MBTL2

**Figure 4:**
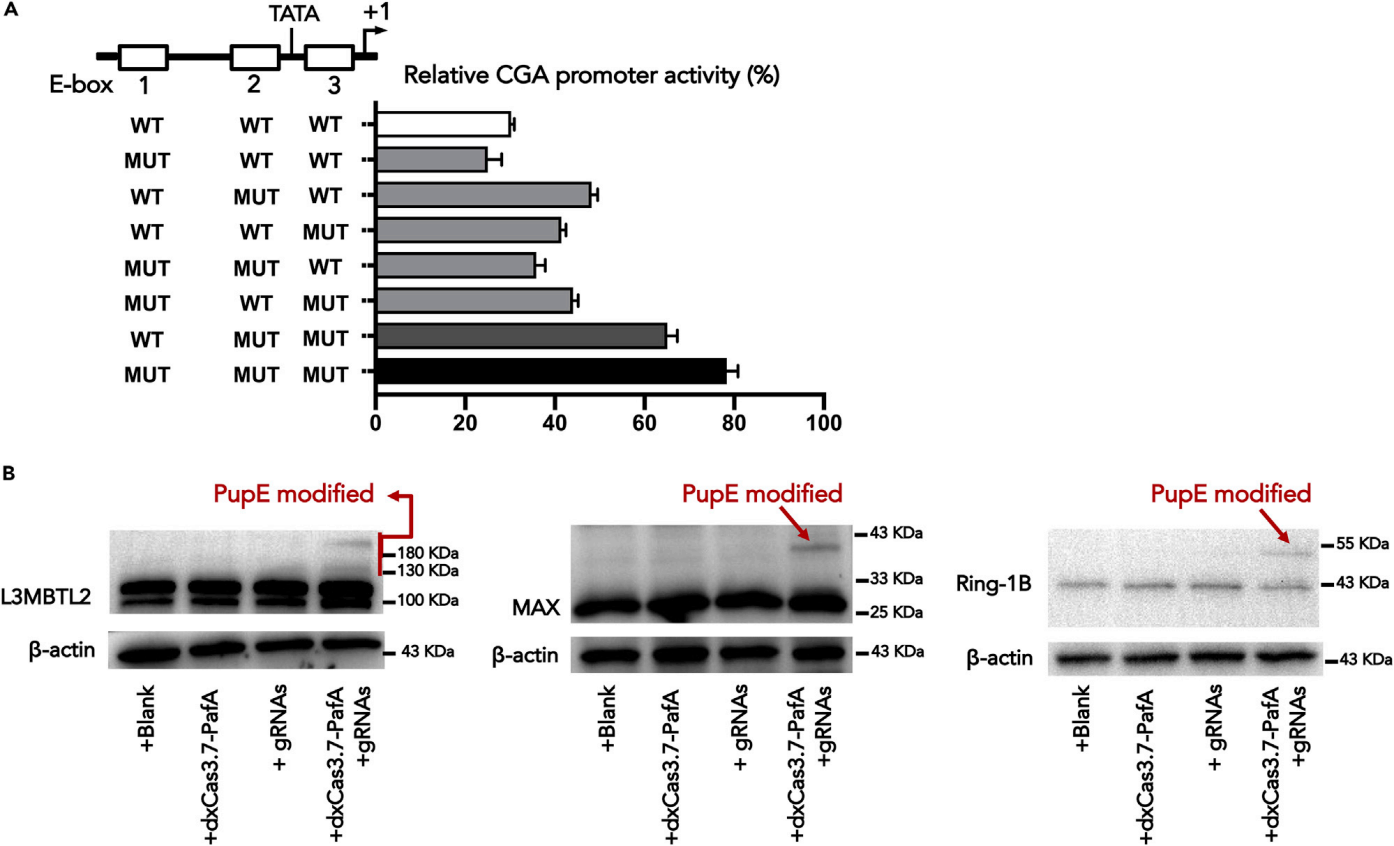
L3MBTL2, MAX, and RING1B synergistically inhibit CGA expression (A) The proximal CGA promoter contains three E-box elements. The plasmid containing the wild-type sequence of the CGA promoter (WT) or mutations in different E-boxes (MUT) was co-transfected with either the pcDNA3.1-L3MBTL2 plasmid or the pcDNA3.1 empty vector. Data are presented as the relative Luc activity of CGA promoters in the presence of exogenous L3MBTL2 as a percentage of the activity of the same reporter in transfections with the control vector. Data are presented as means ± SD (n = 3). (B) *In vitro* Pup(E) modification assay of L3MBTL2 overexpressed cells co-transfected with dxCas3.7-PafA and gRNAs. Western blots showed Pup(E) modification of L3MBTL2, MAX, and RING1B.

We proposed that L3MBTL2 represses CGA by mediating histone modifications; however, the mechanism by which L3MBTL2 targets specific loci is not clear. L3MBTL2 selectively represses specific genes by recruiting the ncPRC1.6 complex to the promoters of its specific target genes. L3MBTL2-dependent ncPRC1.6 binding sites have been reported to be enriched for bHLH E-box motifs (Stielow et al., 2018). L3MBTL2 facilitates PRC1.6 binding site selection by promoting binding of the MGA/MAX heterodimer to the E-box or T-box on the target promoter. To examine this mechanism in L3MBTL2-mediated CGA repression, we mutated three E-boxes of CGA (Figure S3). We compared the ability of L3MBTL2 to repress different reporter constructs carrying different combinations of mutated E-boxes (Figure 4A). The results showed that the three E-boxes cooperate in L3MBTL2-mediated CGA repression and that triple-mutant constructs significantly blocked L3MBTL2-mediated CGA repression, indicating that the E-boxes on the CGA promoter are essential for specific selective repression of CGA by L3MBTL2.

Collectively, L3MBTL2 represses CGA via an H2AK119ub1-dependent mechanism, and the E-box on the CGA promoter determined it to be a specific target of L3MBTL2. Thus, the interaction of L3MBTL2, RING1B, and MAX should play a crucial role in suppressing CGA. To further verify our statement, we applied the PUP-IT (pupylation-based interaction tagging) proximity-tagging system (Liu et al., 2018b) to study the interactome of the CGA promoter. PafA was fused with the CRISPR-dxCas3.7 protein (dxCas3.7-PafA), and gRNAs were designed to lead dxCas3.7-PafA to the position near the TSS sequence of the CGA promoter. In addition, we established a Dox inducible cell line based on L3MBTL2 overexpression cells (PANC1-L3MBTL2-OE -Pup(E)^TET-ON^) for controlling the expression of Pup(E). The PANC1-L3MBTL2-OE-Pup(E)^TET-ON^ cells were co-transfected with dxCas3.7-PafA and gRNAs, followed by dox induction. In this case, the Pup(E)-modified proteins are the ones that interact with the CGA promoter. We observed a high-molecular-weight band in the co-transfection of dxCas3.7-PafA and gRNAs groups, suggesting Pup(E)-modified L3MBTL2, MAX, and RING1B (Figure 4B). By contrast, cells transfected with dxCas3.7-PafA and gRNAs alone lacked the Pup(E)-modified proteins. Therefore, these results reveal that L3MBTL2, MAX, and RING1B are colocalized to the CGA promoter, suggesting that these three proteins may form protein complexes and synergistically inhibit CGA expression. In summary, we propose a mechanism that MGA/MAX assists L3MBTL2 in targeting specific CGA promoters, and L3MBTL2 interacts with ncPRC1.6 core components such as RING1B and HDACs, thereby inhibiting CGA transcription by regulating the histone H2AK119ub1, acetylation (H3Ac and H4Ac), and H3K27me3.

### CGA plays an individual tumor suppressor role in PDAC

**Figure 5:**
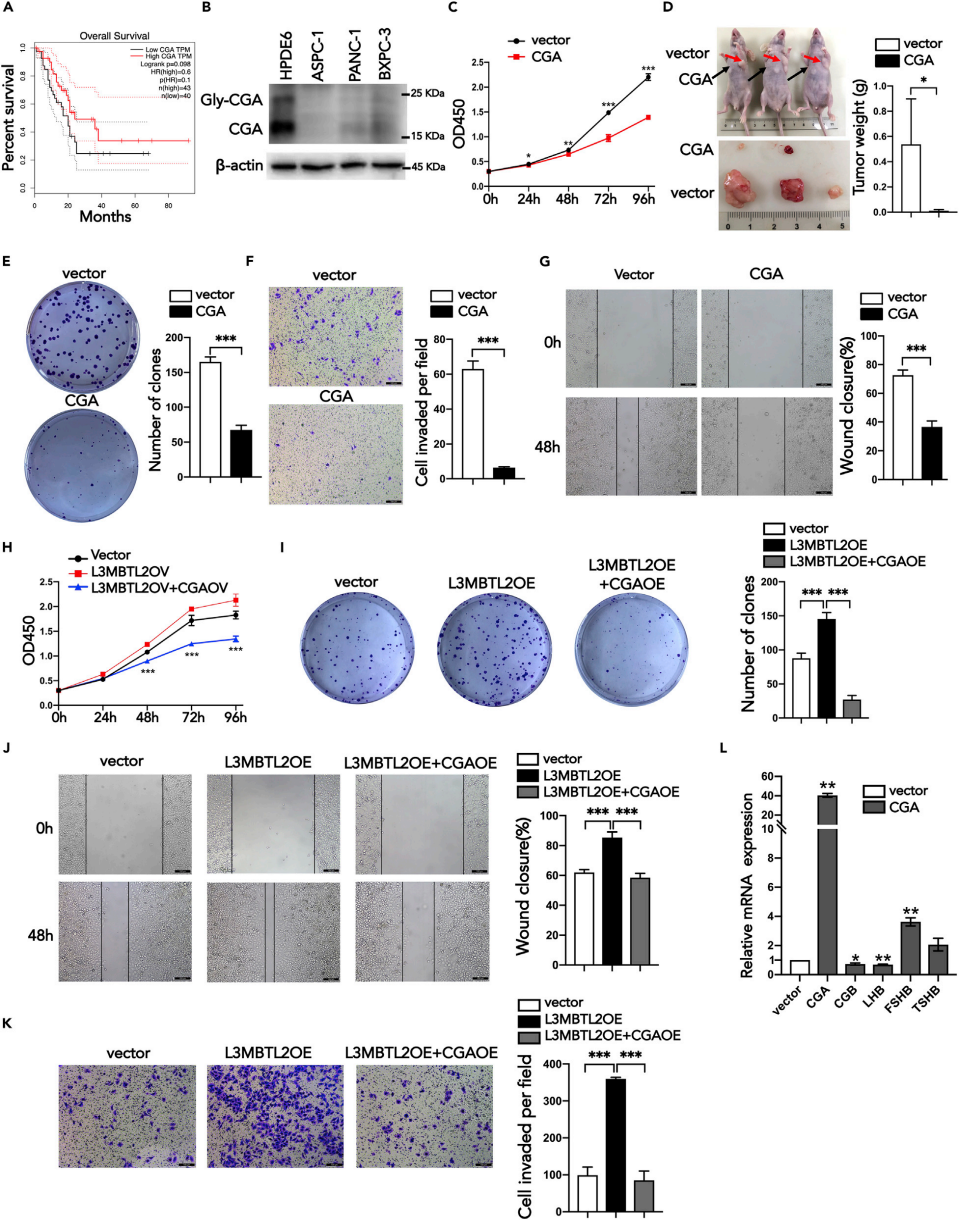
CGA inhibits pancreatic cancer progression (A) The correlations between the expression of CGA and patient survival rate. (B) The protein levels of CGA in pancreatic cancer cells and pancreatic ductal epithelial cells. (C) Cell proliferation of PANC-1 cells expressing exogenous CGA. (D) The volumes and weight of subcutaneous tumors formed by PANC-1 cells expressing exogenous CGA and vectors. (E) Clone forming assay of CGA-overexpressing PANC-1 cells. (F and G) Transwell assays (F) and wound healing assays (G) of PANC-1 cells expressing exogenous CGA and vectors. (H–K) CGA overexpression abolished the promotion of cell proliferation (H), colony formation capacity (I), migration (J) and invasion (K) caused by overexpression of L3MBTL2 in PANC-1 cells. (L) The relative mRNA levels of CGA, CGB, LHB, FSHB, and TSHB in CGA-overexpressing PANC-1 cells. Data are presented as means ± SD (n = 3). ∗p < 0.05, ∗∗p < 0.01, ∗∗∗p < 0.001, t-tests. Scale bars, 100 μm (F, G, J and K).

According to limited clinical evidence, there is no direct evidence for the role of CGA in cancer. According to the TCGA database, a downregulated expression level of CGA was associated with a poor survival rate of patients with PDAC (Figure 5A). To further verify the decreased CGA expression level in PDAC, we analyzed the protein levels of CGA in selected three PDAC cell lines (ASPC-1, PANC-1, and BXPC-3) and one pancreatic ductal epithelial cell line (HPDE6-C7). The western blot detected a significantly decreased CGA expression level in PDAC cells compared to HPDE6-C7 cells, suggesting the tumor suppressor role of CGA in PDAC (Figure 5B). To determine the role of CGA in PDAC and further validate its association with L3MBTL2, we stably overexpressed CGA into PANC-1 cells and into PANC-1 cells overexpressing L3MBTL2. CGA overexpression significantly attenuated cell proliferation, colony formation, migration, and invasive abilities (Figure 5C and 5E–G) compared with the corresponding control. In addition, elevated CGA expression inhibited tumor growth in subcutaneous transplantation models in nude mice (Figure 5D). Simultaneously, we found that the tumor-promoting capacity of L3MBTL2 could be markedly abolished by CGA overexpression (Figures 5H–5K), whereas the loss of CGA could also rescued the antitumor effect of L3MBTL2 knockdown (Figure S5). In summary, these results reveal that the oncogenic function of L3MBTL2 in pancreatic cancer is closely related to its negative regulatory effect on CGA, which plays a tumor suppressor role in PDAC.

Many studies have presented the critical roles of glycoprotein hormones in the diagnosis, monitoring, and treatment of different types of cancer, including breast, gastric, prostate, thyroid, and many other cancers (Kolbl et al., 2018; Crawford and Schally, 2020; Dizeyi et al., 2019; Song et al., 2019; Zhao et al., 2018; Wu et al., 2019). CGA encodes the alpha subunit of glycoprotein hormones, including hCG, LH, FSH, and TSH. Therefore, we wondered whether the role of CGA in PDAC is associated with these four glycoprotein hormones. However, according to the qPCR results, CGA overexpression was not accompanied by overexpression of CGB, LHB, TSHB, or FSHB to produce ectopic hormones in PDAC (Figure 5L). In addition, loss of CGB, LHB, TSHB, and FSHB barely affected the antitumor effect of CGA overexpressing in PANC-1 cells (Figure S6). Therefore, CGA might have an individual role in PDAC and is independent of ectopic glycoprotein hormone production.

### Glycosylated CGA isoform 1 plays a major role in PDAC

**Figure 6:**
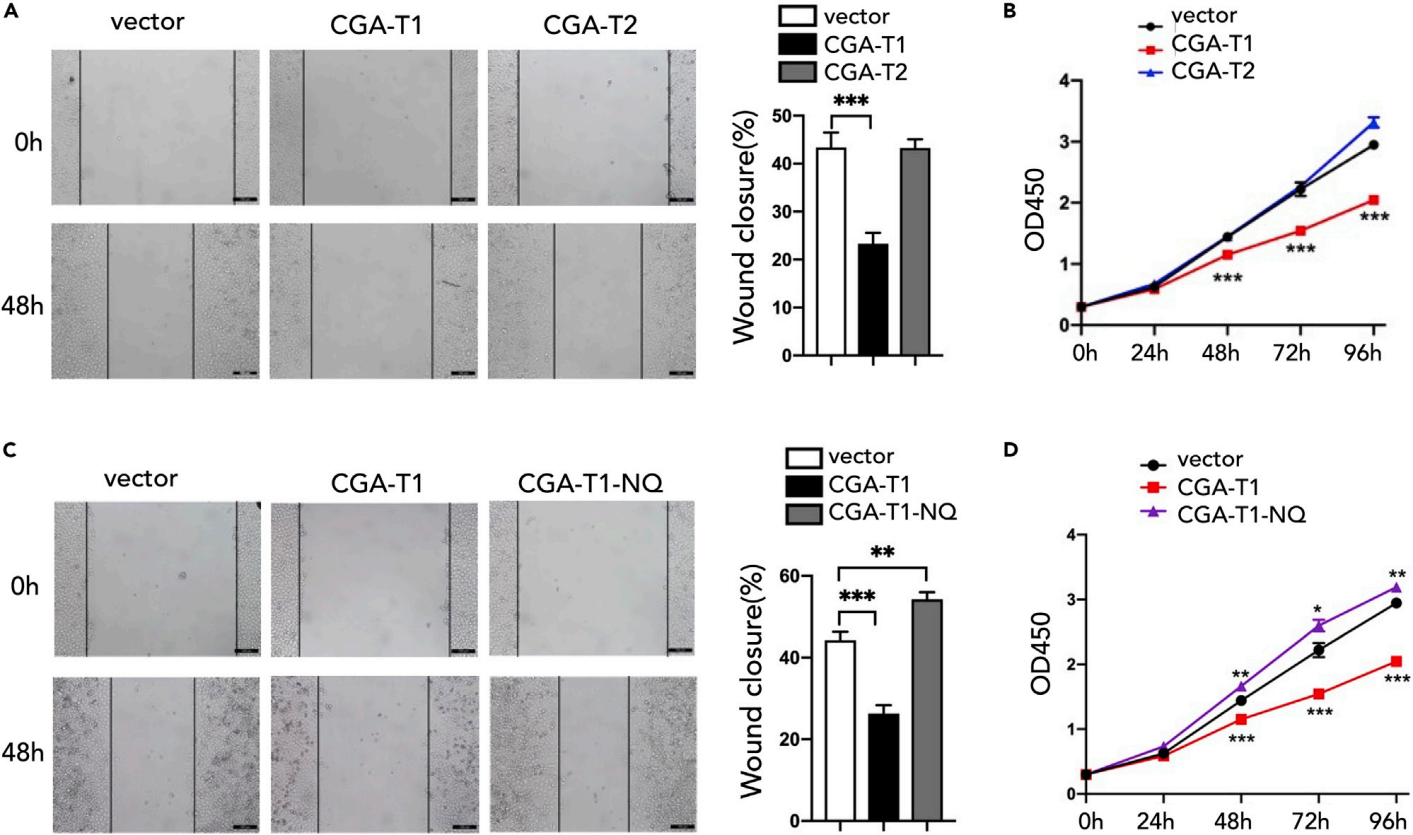
Glycosylated CGA-T1 inhibits pancreatic cancer progression (A and B) wound healing (A) and proliferation assay (B) of PANC-1 cells expressing exogenous vector, CGA-T1, and CGA-T2. (C and D) wound healing (C) and proliferation (D) assays of PANC-1 cells expressing exogenous vector, CGA-T1, and CGA-T1-NQ. Data are presented as means ± SD (n = 3). ∗p < 0.05, ∗∗p < 0.01, ∗∗∗p < 0.001, t-tests. Scale bars, 100 μm (A and C).

Having established the function of CGA in inhibiting tumor progression, we next sought to determine its underlying mechanisms. CGA has two transcripts, and CGA is a glycosylated protein containing two N-glycosylation sites. We wonder about the function of the two transcripts of CGA in PDAC, and whether the tumor suppressive function of CGA is related to protein glycosylation. To test this, we compared the function of four established stable overexpressed cell lines (CGA-T1, CGA-T2, CGA-T1-NQ, and CGA-T2-NQ) in PDAC progression. CGA-T1 was identified as the major transcript that functions in PDAC, which promoted the proliferation and migration of PDAC cells, whereas CGA-T2 barely affected PDAC progression (Figures 6A and 6B). In addition, mutation of N glycosylation sites of CGA-T1 abolished the suppressor function of CGA-T1 (Figures 6C and 6D), indicating that the function of CGA-T1 depends on its own glycosylation. Meanwhile, the CGA-T2 and CGA-T2-NQ cells did not present any function in PDAC (Figure S7). Collectively, the glycosylated CGA isoform 1 plays a major role in PDAC.

### Glycosylated CGA-T1 inhibits autophagy through multiple pathways

**Figure 7:**
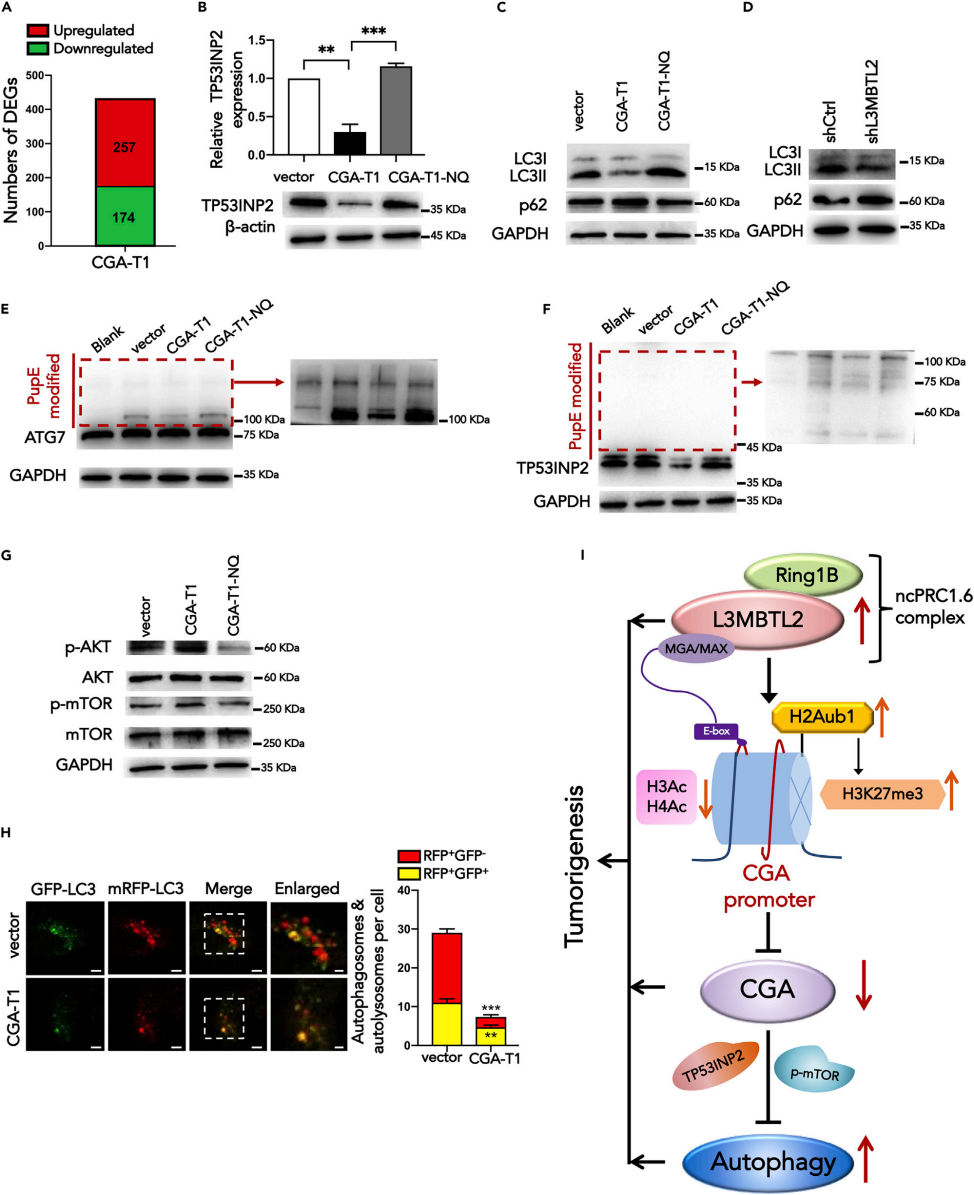
Glycosylated CGA-T1 inhibits autophagy in PDAC (A) Differentially expressed genes with over 2-fold expression changes in PANC-1 cells expressing exogenous CGA-T1 compared with control cells, according to RNA-seq results. (B) The relative mRNA and protein levels of TP53INP2 in PANC-1 cells after CGA-T1 and CGA-T1-NQ overexpression. (C) Comparison of LC3II and P62 expression in PANC-1 cells overexpressed CGA-T1, CGA-T1-NQ, and control vector by Western blot analyses. (D) Western blotting analysis of LC3II and P62 expression in shL3MBTL2 cells. (E and F) *In vitro* PupE modification assay of PANC-1-vector, PANC-1-CGA-T1-OE, and PANC-1-CGA-T1-NQ-OE cells transfected with LC3B-PafA-IRES-PupE vector. PANC-1-vector cells transfected with the PafA-IRES-PupE vector were used as the blank group. Western blots showed PupE modification of ATG7 (E) and TP53INP2 (F), indicating interaction of LC3-ATG7 and LC3-TP53INP2. Because of the difference in protein content, the red box area developed separately and showed multiple modification bands. (G) Western blotting analysis of the p-AKT, AKT, p-mTOR, and mTOR protein expression in PANC-1 cells expressing exogenous vector, CGA-T1, and CGA-T1-NQ. GAPDH served as the internal control. (H) The autophagic flux was detected in indicated cells that were transfected with mRFP-GFP-LC3 plasmid. Scale bar, 5 μm, scale bar in enlarged image, 2 μm. The numbers of red puncta (RFP^+^GFP^−^) versus yellow puncta (RFP^+^GFP^+^) per cell in each cell line were quantified. (I) A Schematic representation of L3MBTL2 interacting with PRC1.6 complexes to mediate the regulation of histone modifications (upregulated H2Aub1 and H3K27me3 and downregulated H3 and H4 acetylation), which synergistically repress CGA transcription. The MAX/MGA heterodimer is required for targeting PRC1.6 to the CGA promoter by binding to its E-boxes. CGA plays an individual tumor suppressor role in PDAC, which is associated with its negative regulation of autophagy through multiple pathways, including PI3K/Akt/mTOR and TP53INP2 pathways. The upregulation of L3MBTL2, L3MBTL2-mediated CGA repression, and suppressed CGA-mediated upregulation of autophagy promotes tumorigenesis in pancreatic cancer. Data are presented as means ± SD (n = 3). ∗p < 0.05, ∗∗p < 0.01, ∗∗∗p < 0.001, t-tests.

To further investigate the mechanism of the individual tumor suppressor role of CGA-T1 in PDAC, we performed RNA-Seq to analyze the gene expression profiles of PANC-1-CGA-T1-OE compared with control cells. We identified 257 upregulated genes and 174 downregulated genes after CGA overexpression (Figure 7A). Among the top 15 enriched KEGG pathways, there were several pathways closely related to tumor development, including focal adhesion, Hippo signaling, PI3K-Akt signaling, and the TGF-beta signaling pathway (Figure S8). These results suggest that CGA has individual biological functions and may therefore be involved in multiple tumorigenesis mechanisms.

Among the DEGs, TP53INP2/DOR (tumor protein p53 inducible nuclear protein 2) is one of the most strongly downregulated genes in response to CGA-T1 overexpression. TP53INP2 has been shown to participate in many cancers and is involved in multiple stages of autophagy. Previous studies show that upon starvation, TP531NP2 interacts with nuclear LC3 and redistributes LC3 by shifting from the nucleus to the cytoplasm, further facilitating autophagosome formation by enhancing the LC3-ATG7 interaction (Huang et al., 2015; You et al., 2019). In addition, the autophagy activation is prevalent in PDAC and promotes the development of pancreatic cancer (Tsang et al., 2020; Yang et al., 2011; Li et al., 2016). We speculated that the role of CGA-T1 in PDAC might be associated with the TP53INP2-mediated autophagy process. To test our hypothesis, we confirmed the significant negative regulation of TP53INP2 by CGA-T1 (Figure 7B) and subsequently investigated the effect of CGA-T1 on autophagy. According to western blot assays, the expression levels of LC3II decreased markedly in CGA-T1 overexpressed PANC-1 cells, whereas p62 protein expression was significantly increased compared with the control cells (Figure 7C). To confirm the effect of CGA-T1 on autophagy, an mRFP-GFP-LC3B construct was used to detect the autophagic flux. As shown in Figure 7H, immunofluorescence assay revealed a significant decrease in both autophagosome and autolysosome in CGA-T1-overexpressed cells (Figure 7H). These results indicated that CGA-T1 inhibited the autophagy process. Considering the L3MBTL2 strongly suppresses transcription of CGA, we also verified the inhibitory effect of shL3MBTL2 on autophagy (Figures 7D and S11). Notably, the reverse trend was observed in CGA-T1-NQ cells, where overexpression of CGA-T1-NQ increased LC3II expression and autophagosomes, which promoted autophagy (Figure 7C). The results suggested that CGA-T1 prevents the autophagy depending on its own glycosylation. These results are consistent with our previous conclusion that glycosylated CGA-T1 plays a major role in PDAC.

Furthermore, the immunofluorescence assay showed that LC3 distribution in CGA-T1-overexpressed cells was largely enriched in the nucleus (Figure 7H), suggesting that CGA-T1 might influenced the translocation efficiency of LC3 from the nucleus to the cytoplasm. This might be because of CGA-T1 preventing TP53INP2 from assisting LC3 movement from the nucleus to the cytoplasm. Subsequently, we confirmed the LC3-TP53INP2 and LC3-ATG7 interaction in PANC-1 cells. In addition, the decreased PupE-modified bands indicated that CGA-T1 overexpression reduced the LC3-TP53INP2 and LC3-ATG7 interaction (Figures 7E and 7F). The decreased interaction can be explained as CGA overexpression leads to reduced TP53INP2 expression, decreasing participation in LC3-TP53INP2 interactions, and diminishing the promotion effect of TP53INP2 on LC3-ATG7 interaction. These results support our hypothesis that glycosylated CGA-T1 inhibits autophagy partially through the TP53INP2-mediated pathway.

Although TP53INP2 plays an important role in the early stage of autophagy, TP53INP2 alone is not enough to support the significant inhibition of CGA on autophagy. Therefore, we suspect that the effect of CGA on autophagy is not limited to the regulation of TP53INP2. According to the RNA-seq results, we found that among the enriched KEGG pathways (Figure S6), PI3K-Akt-mTOR signaling pathway is frequently activated and plays an important role in the pathogenesis of PDAC (Ebrahimi et al., 2017). As a critical modulator of autophagy, PI3K/AKT/mTOR-mediated autophagy is involved in many cancers, including PDAC (Ebrahimi et al., 2017; Liu et al., 2018a; Xu et al., 2020; Zhang et al., 2019). Therefore, we assumed that CGA-T1 might influence autophagy partially by regulating the PI3K/AKT/mTOR-mediated autophagy process. Afterward, we investigated the effect of CGA-T1 on the central checkpoints of PI3K-Akt-mTOR-mediated autophagy, mTOR. According to western blot assays, CGA-T1 overexpression upregulated the expression levels of phospho-Akt (p-Akt) and phospho-mTOR (p-mTOR), whereas CGA-T1-NQ had no effect (Figure 7G). Furthermore, we used the mTOR inhibitor Rapamycin (Rap), confirmed the autophagy promoted by Rap alone in PDAC, and verified the relationship between CGA and the mTOR pathway in autophagy (Figure S9). These results indicated that glycosylated CGA-T1 inhibits the initiation of autophagy partially through the PI3K/Akt/mTOR pathway. Besides, we do not exclude CGA-T1 inhibiting autophagy through other mechanisms, such as modulates other key proteins, and further studies are needed. Collectively, we demonstrated that the tumor suppressor role of glycosylated CGA-T1 in PDAC is associated with its negative regulation of autophagy through multiple pathways, including PI3K/Akt/mTOR and TP53INP2 pathways.

### L3MBTL2 plays oncogenic roles in lung and hepatology carcinoma

Data from the GEPIA database indicated that L3MBTL2 mRNA levels are significantly upregulated in various types of cancers, including lung cancer, liver cancer, breast cancer, skin cutaneous melanoma, and stomach adenocarcinoma (Figure S1). To explore the function of L3MBTL2 in other cancers, we established L3MBTL2 knockdown stably transfected H1299 and HepG2 cell lines. L3MBTL2 knockdown significantly decreased cell proliferation, migration, and invasion in H1299 (Figures S10A–S10C) and HepG2 (Figures S10D–S10F) cells. These data suggest that L3MBTL2 plays an oncogenic role in lung and hepatology carcinoma and it may also be critical for other cancers.

## Discussion

L3MBTL2 is a crucial component of a noncanonical PRC1 subgroup termed polycomb repressive complex 1.6 (PRC1.6), and it has been implicated in transcriptional repression and chromatin compaction (Gao et al., 2012). However, the mechanism by which L3MBTL2 represses its target genes is still unclear, and its biological functions are largely undefined. Our study shows for the first time that L3MBTL2 functions as an oncogene, significantly promoting cell proliferation, migration, invasion, and tumorigenesis in pancreatic cancer. We also investigated the effects of L3MBTL2 in liver (HepG2) and lung (H1299) cancer cells, indicating a critical oncogenic role for L3MBTL2 in tumor initiation and progression. The effect of L3MBTL2 may involve transcriptional repression of the expression of the downstream target gene CGA, and the E-box of CGA plays a critical role in specific selective repression of CGA by L3MBTL2. Mechanistically, we suggested that L3MBTL2 represses CGA mainly by regulating H2AK119ub1, whereas changes in acetylation (H3Ac and H4Ac) and H3K27me3 may contribute to optimal L3MBTL2-mediated repression of CGA. In addition, we demonstrated CGA acts as a tumor suppressor gene in PDAC and verified that the oncogenic function of L3MBTL2 is closely related to its regulatory effect on CGA. Furthermore, we also explored the mechanism of CGA in PDAC, which is glycosylated CGA-T1 inhibits multiple stages of autophagy through multiple pathways. Thus, we propose the L3MBTL2/CGA pathway in PDAC, which may represent a potential therapeutic strategy for treatment.

Polycomb group (PcG) proteins assemble into two major repressive complexes—PRC1 and PRC2—that catalyze two repressive histone modifications: H2AK119ub1 and H3K27me3. The canonical view suggests that H3K27me3 is required for the recruitment of PRC1 to genomic sites (Simon and Kingston, 2009). However, recent studies have suggested that H2AK119ub1 deposition by noncanonical PRC1 facilitates the recruitment of PRC2 and downstream H3K27me3 (Blackledge et al., 2014; Cooper et al., 2014). L3MBTL2 is a component of the ncPRC1.6 complex, which also contains other core components, such as MGA, RING1B, HDAC1/2, PCGF6, and E2F6. In this study, ChIP-qPCR analysis revealed that chromatin modifications, including H2AK119ub1, acetylation, and H3K27me3, at the CGA promoter were changed after overexpression of L3MBTL2, which might be associated with the interaction between L3MBTL2 and the associated enzymes (RING1B and HDAC1). Moreover, our data indicated that L3MBTL2-mediated CGA repression was significantly abolished by a loss of H2AK119ub1 deposition at the CGA promoter, but it was partially rescued by increased histone acetylation. In addition, the decreased H3K27me3 at the CGA promoter did not impact CGA transcription, suggesting that L3MBTL2-mediated CGA repression is H3K27me3 independent. Our study is consistent with a previous study claiming that ncPRCs target chromatin by H3K27me3-independent mechanisms (Blackledge et al., 2014). Collectively, we demonstrated that L3MBTL2 represses CGA transcription mainly by regulating H2AK119ub1, whereas changes in acetylation (H3Ac and H4Ac) and H3K27me3 may contribute to optimal L3MBTL2-mediated repression of CGA. It will be of interest to determine whether this represents a general mechanism by which L3MBTL2 represses target genes.

L3MBTL2 mediates transcriptional repression of specific target genes. However, the mechanism by which L3MBTL2 targets specific loci remains unclear. A recent study indicated that L3MBTL2-mediated genomic targeting is closely related to its interaction with the PRC1.6 complex. L3MBTL2 facilitates PRC1.6 binding site selection by promoting and stabilizing the binding of the MGA/MAX heterodimer to the E-box or T-box in the target sequence (Stielow et al., 2018). In our study, we confirmed the critical role of the E-box in the CGA promoter in specific selective repression by L3MBTL2. Cells transfected with the reporter constructs carrying triple-mutant E-boxes significantly blocked L3MBTL2-mediated CGA repression. L3MBTL2 interacts with the core components of the PRC1.6 complex, making it critical for transcriptional repression and genomic targeting of PRC1.6. Indeed, we applied the PUP-IT proximity-tagging system to identify the proteins that interacted with the CGA promoter. The results showed that L3MBTL2, MAX, and RING1B are all localized to the CGA promoter. However, other mechanisms that may determine genomic targeting and transcriptional repression still need to be further investigated.

CGA encodes the alpha subunit of glycoprotein hormones that includes hCG, LH, FSH, and TSH. However, in this study, we demonstrated that CGA plays a role as a tumor repressor that is independent of the hormones it is typically associated with in PDAC. CGA overexpression significantly inhibited the proliferation rate and attenuated the migration and invasive abilities of PDAC cells. In addition, we found no correlation between CGB, LHB, TSHB, or FSHB mRNA levels and CGA mRNA overexpression. Thus, we propose that CGA plays an individual role and is independent of ectopic glycoprotein hormone production. However, the underlying mechanism is still unclear. Interestingly, we found the two transcripts of CGA have different functions, and the tumor suppressive function of CGA is related to the protein glycosylation. In two of the CGA transcripts, CGA-T1 was identified as the primary transcript that functions in PDAC, whereas mutation of N glycosylation sites of CGA-T1 abolished the suppressor function of CGA-T1, indicating that the tumor suppressor function of CGA-T1 depends on its own glycosylation. Collectively, the function of CGA in PDAC depends on its alternative pre-mRNA splicing and protein glycosylation. In addition, we found that the shL3MBTL2 upregulated the transcription of total CGA and also promoted the glycosylation of CGA. We speculated that L3MBTL2 might transcriptionally repress glycotransferase or have other complex biological functions that influence the CGA glycosylation process, which need to be studied further.

In the further mechanism study, we found that CGA-T1 inhibited autophagy, characterized by decreased LC3-II, upregulated p62 expressions, and decreased autophagosomes in the cytoplasm. In addition, glycosylated CGA-T1 negative regulated the TP53INP2 expression and reduced the LC3-TP53INP2 and LC3-ATG7 interaction, suggesting that CGA-T1 inhibits the autophagy through TP53INP2 pathway. Meanwhile, we found that CGA-T1-NQ promoted autophagy, while barley affecting TP53INP2 expression level. Therefore, we suspect that the effect of CGA on autophagy is not limited to a single stage. Indeed, we observed that glycosylated CGA-T1 enhanced the p-AKT and p-mTOR expression, indicating that CGA-T1 may inhibit the initial stage of autophagy. Therefore, the inhibition effect of CGA-T1 on autophagy might involve multiple stages; however, the specific molecular mechanism needs to be further studied. Moreover, our study demonstrated that the glycosylated CGA-T1 plays a major role in PDAC. Thus, the correlation between CGA glycosylation and autophagy also needs to be studied in the future. In addition, glycoprotein hormones have been demonstrated to play a role in many types of cancers. It is worth knowing whether CGA exhibits an independent tumor suppressor effect in other cancers. If CGA plays a universal role in tumor suppression, it will provide a new strategy and therapeutic targets for cancer treatment.

In summary, our study revealed a distinct oncogenic role of L3MBTL2 in tumor development and an L3MBTL2-mediated transcriptional repression mechanism. We demonstrated that L3MBTL2 interacted with ncPRC1.6 core components to repress downstream CGA via an H2AK119ub1-dependent mechanism, and the E-box on the CGA promoter determined it to be a specific target of L3MBTL2. We further validated that CGA plays an individual tumor suppressor role in pancreatic cancer. Indeed, CGA-overexpressing cells showed attenuated proliferation, migration, and invasive abilities, which were independent of glycoprotein hormones. Moreover, CGA-T1 was identified as the major transcript in PDAC, and the tumor suppressor function of CGA-T1 depends on its own glycosylation. Furthermore, glycosylated CGA-T1 inhibited PDAC might be partly through negative regulated autophagy through multiple pathways, including PI3K/Akt/mTOR and TP53INP2 pathways (Figure 7I). The discovery of the L3MBTL2/CGA axis and its impact on autophagy and PDAC progression provides a way to explore more efficient therapeutic strategies.

### Limitations of the study

Despite detecting a significant transcriptional suppression of CGA by L3MBTL2, we proposed the mechanism by mediating histone modifications, which is closely related to the ncPRC1.6 complex in which L3MBTL2 embedded. However, the mechanism by which L3MBTL2 collaborates with PRC1.6 members to rapidly and selectively target specific genes needs to be further explored. In addition, we found that the shL3MBTL2 upregulated the transcription of total CGA and also promoted the glycosylation of CGA. We speculated that L3MBTL2 might transcriptionally repress glycotransferase or have other complex biological functions that influence the CGA glycosylation process, which need to be studied further. Moreover, we showed the antitumor function and regulatory mechanism of CGA mainly in PDAC cell lines; however, CGA is a secreted protein. Thus, the function of secreted CGA and its influence on function of cellular CGA are still unclear, and more *in vivo* experiments are needed. Furthermore, we observed that glycosylated CGA-T1 showed opposite effects on the autophagy process compared with CGA-T1-NQ cells. Therefore, the correlation between CGA glycosylation and autophagy might be a critical element and needs to be studied in the future.

## STAR★Methods

### Key resources table

**Table:** 

REAGENT or RESOURCE	SOURCE	IDENTIFIER
**Antibodies**		

L3MBTL2	Biorbyt	Cat#: orb411730; RRID: AB_10932814
CGA (HCG Alpha Polyclonal Antibody)	Proteintech	Cat#: 25014-1-AP
TP53INP2	Abcam	Cat#: ab273012
ATG7	Abcam	Cat#: ab52472
LC3B	Abcam	Cat#: ab192890; RRID: AB_2827794
SQSTM1/p62	Cell Signaling Technology	Cat#: 5114S; RRID: AB_10624872
mTOR	Cell Signaling Technology	Cat#: 2983S; RRID: AB_2105622
p-mTOR	Cell Signaling Technology	Cat#: 5536S; RRID: AB_10691552
AKT	Cell Signaling Technology	Cat#: 4685S; RRID: AB_2225340
p-AKT	Cell Signaling Technology	Cat#: 4060S; RRID: AB_2315049
GAPDH	Cell Signaling Technology	Cat#: 5174S; RRID: AB_10622025
Beta-Actin	Biorbyt	Cat#: orb181785
H2AK119ub1	Cell Signaling Technology	Cat#: 8240S; RRID: AB_10891618
H3K27me3	Active-Motif	Cat#: 39055; RRID: AB_2561020
HDAC1	Active-Motif	Cat#: 40967; RRID: AB_2614948
H3K9me3	Active-Motif	Cat#: 39065; RRID: AB_2793334
H3K36me3	Active-Motif	Cat#: 61902; RRID: AB_2615073
Goat Anti-Rabbit IgG antibody (HRP)	Biorbyt	Cat#: orb43514; RRID: AB_10998428
Goat Anti-Mouse IgG(H+L) antibody (HRP)	Biorbyt	Cat#: orb506151
Rabbit mAb IgG	Cell Signaling Technology	Cat#: 66362

**Chemicals, peptides, and recombinant proteins**		

RPMI-1640	Gibco	Cat#: A1049101
DMEM	Gibco	Cat#: 11995040
EBSS	Gibco	Cat#: 14155063
Fetal bovine serum (FBS)	Gibco	Cat#: 10099141
PBS	Gibco	Cat#: 70011-044
Streptomycin reagent	Hyclone	Cat#: SV30010
Penicillin	Hyclone	Cat#: SV30010
TRIzol Reagent	Invitrogen	Cat#: 15596026
Cell lysis buffer	Beyotime	Cat#: P0013
Protease and phosphatase inhibitor cocktail	Beyotime	Cat#: P1050
RIPA	Beyotime	Cat#: P0013B
Crystal violet	Sigma	Cat#: C6158
pentobarbital sodium	Sigma	Cat#: P3761
Rapamycin	MedChemExpress	Cat#: HY-10219
Skim milk	BD	Cat#: 232100

**Critical commercial assays**		

HiScript III 1st Strand cDNA Synthesis Kit	Vazyme	Cat#: R312-01/02
ChamQ SYBR Color qPCR Master Mix	Vazyme	Cat#: Q411-02/03
BeyoECL Moon super sensitivity detection kit	Beyotime	Cat#: P0018FS
Chromatin IP DNA Purification Kit	Active Motif	Cat#: 58002
CUT&RUN Assay Kit	Cell Signaling Technology	Cat#: 86652
Cell Counting Kit-8	Solarbio	Cat#: CK04

**Deposited data**		

Raw and analyzed RNA sequencing data	This paper	GEO: GSE184834

**Experimental models: Cell lines**		

Human: PANC-1	ATCC	Cat#: CRL-1469
Human: ASPC-1	ATCC	Cat#: CRL-1682
Human: BXPC-3	ATCC	Cat#: CRL-1687
Human: HPDE6-C7	ATCC	RRID: CVCL_0P38
Human: H1299	ATCC	Cat#: CRL-5803
Human: HepG2	ATCC	Cat#: HB-8065

**Experimental models: Organisms/strains**		

Mouse: BALB/c nude mice (6-week-old, female)	Charles River	Cat# 401

**Oligonucleotides**		

Sh L3MBTL2-1 5’- GCTTCGGTATGAAGGCTTTGAA-3’	This paper	N/A
Sh L3MBTL2-2 5’- GCGTGTGAAGGAAGAGCATCTA-3’	This paper	N/A
Sh CGA-1 5’- CCACTGCAGTACTTGTTATTA -3’	This paper	N/A
si-CGB	Thermo Fisher Scientific	Cat#: 247166
si-LHB	Thermo Fisher Scientific	Cat#: 214223
si-TSHB	Thermo Fisher Scientific	Cat#: 138744
si-FSHB	Thermo Fisher Scientific	Cat#: 144547
RT-PCR primers (Table S1)	This paper	N/A
CGA-promotor-sgRNA1 5’-CATGGTAAAAATTGACGTCA-3’	This paper	N/A
CGA-promotor-sgRNA2 5’- ACCAAGTACCCTTCAATCAT- 3’	This paper	N/A
CGA-promotor-sgRNA3 5’- TGATCCCAGGGCTTAGATGC-3’	This paper	N/A

Recombinant DNA		

pCDH-EF1α-MCS-T2A-Puro	System Biosciences	CD527A-1
pLVX-shRNA2	Clonetech	632179
pGL4.11	Promega	E6661
pcDNA3.1	Thermo Fisher Scientific	V79520
pLVX-TetONE-Puro	Takara	631849
dxCas(3.7)-VPR	Addgene	108383
ptfLC3	Addgene	21074
pEF-PUP-IT (pupylation-based interaction tagging) proximity-tagging system	(Liu et al., 2018b)	N/A
pLVX-Pup(E)-TET-ONE	This paper	N/A
pGL4-CGApromoter	This paper	N/A
pcDNA3.1-dxCas9(3.7)-PafA	This paper	N/A
pcDNA3.1-LC3B-PafA-IRES-PupE	This paper	N/A
pcDNA3.1-dCas9-USP16	This paper	N/A
pcDNA3.1-dCas9-KDM6B	This paper	N/A
pcDNA3.1-dCas9-HDAC3	This paper	N/A
pcDNA3.1-dCas9-EP300	This paper	N/A

**Software and algorithms**		

Prism8	GraphPad	https://www.graphpad.com/scientific-software/prism/
SnapGene 4.3	SnapGene	https://www.snapgene.com

### Resource availability

#### Lead contact

Further information and requests for resources and reagents should be directed to and will be fulfilled by the lead contact, Hua Huang (huangh@bjut.edu.cn).

#### Materials availability

This study did not generate new unique reagents.

### Experimental model and subject details

#### Cell lines

Human Pancreatic cancer cell lines (PANC-1, ASPC-1, BXPC-3), human pancreatic ductal epithelial cell line (HPDE6-C7), human hepatology cell line (HepG2) and lung cancer cell line (H1299) were all obtained from ATCC. The H1299 cells were cultured in RPMI-1640 supplemented with 10% FBS (Gibco:10099141), and other cells were maintained in Dulbecco’s modified Eagle medium (DMEM, Gibco:11995040) supplemented with 10% FBS, 100 ng/mL streptomycin reagent (Hyclone, Logan, UT, USA) and 100 U/mL penicillin (Hyclone, Logan, UT, USA). The culture conditions were maintained in a humidified incubator with 5% CO_2_ level at 37°C.

#### Animal studies

Six-week-old female Balb/c nude mice were purchased from Charles River (Beijing, China) and were housed under a 12-h dark, and 12-h light conditions and a standard temperature of 18–23°C with humidity of 40–60%. For *in vivo* tumorigenicity assays, PANC-1 cells (L3MBTL2-OE, sh L3MBTL2, CGA-OE and the corresponding control cells) in 200 μL PBS were subcutaneously injected into the right and left flanks of mice with cell density of 2×10^6^ cells per site. Following 5–6 weeks growth, the mice were anesthetized with 1% pentobarbital sodium (45 mg/kg, intraperitoneal) and subsequently euthanized by cervical dislocation. The tumor weight was measured and the volume was calculated at the ending point. All animal experiments in this study were approved by the Ethics Committee of Beijing University of Technology (protocol number HS202105004).

### Method details

#### Quantitative real time-PCR

Cells were harvested, and TRIzol® Reagent (Invitrogen, USA) was employed to extract total RNA following manufacturer’s instructions. Reverse transcription was performed using the HiScript III 1st Strand cDNA Synthesis Kit (Vazyme Biotech Co., Nanjing, China) according to the manufacturer's protocol. Gene expression was evaluated by qPCR using the ChamQ SYBR Color qPCR Master Mix (Vazyme Biotech Co., Nanjing, China) and run on the ABI STEPONE system (Applied Biosystems, Foster City, CA, USA). The thermocycling protocol included an initial denaturation step at 95°C for 3 min, followed by 40 cycles of denaturation at 95°C for 15 sec, annealing at 60°C for 15 sec and extension at 72°C for 30 sec. The 2-ΔΔCq method was calculated to analyze the relative changes in gene expression according to the previous reported method (Livak and Schmittgen, 2001). β-Actin was used as an internal control. Primers used in this study are listed in Table S1.

#### RNA sequencing and data analysis

The PANC-1-shL3MBTL2, PANC-1-shCtrl, PANC-1-CGA-T1 and PANC-1-vector cells were harvested and performed RNA-seq. The RNA-seq was performed as previous reported (Khan et al., 2022). The RNA-seq data were submitted to Gene Expression Omnibus (GEO): GSE184834.

#### Western blotting

Proteins were extracted using cell lysis buffer RIPA (Beyotime, Shanghai, China) and a protease and phosphatase inhibitor cocktail (Beyotime, Shanghai, China). A total of 20 μg protein/per lane was separated by 4–20% SDS-PAGE gels and subsequently transferred onto a membrane and blocked with 5% skim milk (Difco; BD Biosciences) at room temperature for 2 h. The membranes were then subjected to immunoblotting with appropriate primary at 4°C overnight and secondary antibodies for 2 h at room temperature. Luminescence was visualized using a BeyoECL Moon super sensitivity detection kit (Beyotime, Shanghai, China) according to the manufacturer's protocol.

#### Lentivirus transfection

The the coding DNA sequences (CDSs) of human L3MBTL2 and CGA were subcloned into the pCDH-EF1α-MCS-T2A-Puro lentivirus vector (System Biosciences: CD527A-1)). The shRNA sequences of L3MBTL2, CGA and their negative control were cloned into the pLVX-shRNA2-BSD lentivirus vector upgraded by pLVX-shRNA2 (Clonetech: 632179) (in which the ZsGreen gene sequence is replaced with a BSD resistance gene sequence). Each of the purified plasmids was packaged into a lentivirus and then transfected into PANC-1, ASPC-1, HepG2 and H1299 cells. The lentivirus packing and transfection methods are same as previously reported (Huang et al., 2022). Then, positive overexpressed and knockdown cells were selected using 2 μg/mL puromycin or 5 μg/mL blasticidin S for 10 days.

#### Promoter luciferase reporter assay

A promoter sequence 500 bp in length, ranging from −406 base site to the +94 base site of the CGA gene, were copied into the modified pGL4.11[luc2P] (Promega, Madison, WI) vector. The SV40 promoter, coupled with the Renilla luciferase gene coding sequence, was added to the pGL4.11[luc2P] vector. Cells were cotransfected with the pcDNA3.1-L3MBTL2 plasmid or pcDNA3.1 empty vector and 50 ng of the pGL-CGA-promoter vector. Firefly luciferase activity and the Renilla luciferase signal were measured 48 h after transfection using the Dual-Luciferase Reporter Assay Kit (Promega, Madison, USA) according to the manufacturer’s instructions.

#### Mutant CGA reporter constructs

Three 5’-CANNTG (one 5’-CACCTG and two 5’-CAGGTG) sequences of E-box were mutated to 5’-AANNTA respectively. The mutant reporter constructs used carry the CGA promoters with combinations of the three mutated core sequences. Each combination of mutations was confirmed by sequencing.

#### Cell nucleofection

L3MBTL2-OE/KD adherence cells were digested, washed 3 times with PBS buffer and resuspended with 100 μL room temperature Entranster-E (Nucleofection regent, Engreen Biosystem, Beijing, China) contains 6 μg plasmid (4 μg pcDNA3.1-dCas9-Vector/USP16/HDAC3/EP300/KDM6B plasmid and 2 μg gRNA donor plasmid). The mixture was transferred to the shock cup and nucleofection was performed using Nucleofector 2b Device (Nucleofection system, Lonza, Basel Switzerland). After the program is finished, cells were transferred to the 6-well plates with 1.5 mL culture medium.

#### Detection of protein complexes on CGA promoters

The Biotin-Pup(E) was subcloned into the Tet-On lentivirus vector which contains blasticidin S resistance. The plasmid was packaged into a lentivirus and then transfected into L3MBTL2-OE PANC-1 cells for 48 hours. Then, positive PANC1-L3MBTL2-OE -Pup(E)TET-ON cells were selected using 5 μg/mL blasticidin S for 7 days. Afterwards, a CRISPR-dxCas3.7-based fusion system was established by fusion PafA to the C-terminal tail of full-length dxCas3.7 by a flexible GSG connection linker (dxCas3.7-PafA). The PANC1-L3MBTL2-OE -Pup(E)TET-ON cells were applied to nucleofection with 4 μg dxCas3.7- PafA plasmids and 2 μg gRNA donor plasmid according to the nucleofection method mentioned previously. After 24 hours, 2 μg/ml doxycycline (Dox) was used for another 24 h for Pup(E) induction. The gRNA sequences for targeting CGA promoter are listed in the key resources table.

#### Detection of interaction of LC3 with ATG7 and TP53INP2

LC3B coding sequences was subcloned to the transient vector fused with PafA coding sequence, and the downstream PupE sequence was connected to PafA via an IRES element. PANC1-CGA-T1-OE, PANC1-CGA-T1-NQ-OE and the corresponding control cell line were applied to nucleofection with 4 μg LC3B-PafA-IRES-PupE plasmids according to the nucleofection method mentioned previously. After 36 hours, cells were collected for protein purification and western-blot detection with corresponding antibodies.

#### Chromatin Immunoprecipitation (ChIP)

To perform ChIP experiments, we utilized PANC-1 cells transfected with L3MBTL2 or control vector. 2×10^5^ cells were harvested for each antibody reaction and additional same amount cells for input samples and negative control Rabbit (DA1E) mAb IgG. The CUT&RUN and ChIP assays was performed according to the instruction (CUT&RUN Assay Kit, Cell Signaling Technology, USA). Lyse the cells and fragment the chromatin by sonicating the input samples using M220 focused-ultrasonicator (Covaris, USA) device. The microTUBE-50 AFA Fiber Screw-Cap (Covaris, USA) was used for sample holder and 150 bp target base pair peak program was chosen for chromatin fragmentation. The peak incident power was 75 W and cycles per burst was set to 200 cpb with the total treatment time was 520s. DNA were purified from input and enriched chromatin samples using the Chromatin IP DNA Purification Kit (Active Motif, USA). Quantitative PCR reactions were performed using the ChamQ SYBR Color qPCR Master Mix (Vazyme, Nanjing, China) on the ABI STEPONE system (Applied Biosystems, Foster City, CA, USA). The antibodies used in this procedure are listed in key resources table and the primers used are listed in Table S1. All ChIP assays were repeated three times and individual qPCR reactions performed in triplicates with results presented as mean ± SD.

#### Cell proliferation assay

The Cell Counting Kit-8 (CCK-8) assay was used to evaluate cell proliferative potential. Cells were seeded and cultured in 96-well plates at a cell density of 1,500 cells per well. After adhesion, 10 μL CCK-8 (Cell Counting Kit-8, Solarbio, Beijing, China) reagent was added to each well for 2 extra hours incubation and the absorbance was measured at 450 nm using a Microplate reader (Bio-Rad Laboratories, Inc.).

#### Colony formation assay

Cells were seeded in 6-well plates (300 cells/ well) and incubated in a humidified air containing 5% CO_2_ at 37°C for 10–14 days. Cells were washed with PBS, fixed with 4% paraformaldehyde for 15 min and stained with crystal violet for 15 min at room temperature. After staining, images were captured and the number of colonies (containing cells >50 cells) was counted manually.

#### Wound healing assay

A total of 2×10^5^ cells/well were seeded and cultured in 12-well plates to create a confluent monolayer. After adhesion, a horizontal scratch was made using a sterile 200-μL microliter pipette tip. Cells were cultured for 48 h in a medium containing 2% (wt/vol) FBS, and images of the “scratch closure” were photographed with a light microscope (Leica Microsystems, Inc.).

#### Transwell invasion assay

For transwell invasion assay, a total of 1×10^4^ cells in serum-free DMEM medium were seeded in the upper chamber pre-coated with Matrigel (8 μm Transwell inserts, BD Biosciences). The lower chambers were filled with DMEM with 10% FBS as an attractant. After 24 hours of incubation, cells on the bottom surface were fixed with 4% paraformaldehyde for 15 min and stained using 0.1% crystal violet for 15 min at room temperature. Stained cells were visualized under a light microscope (Leica, USA). For each insert, at least three selected fields were photographed and counted.

#### *In vivo* tumorigenicity assays

Six-week-old female Balb/c nude mice were purchased from Weitonglihua Company (Beijing, China) and were housed under a 12-h dark, and 12-h light conditions and a standard temperature of 18–23°C with humidity of 40–60%. All animal experiments in this study were approved by the Ethics Committee of Beijing University of Technology. L3MBTL2-OE or sh L3MBTL2 and the corresponding control cells (2×10^6^ cells in 200 μL PBS) were subcutaneously injected into the right and left flanks of mice. Following 5–6 weeks growth, the mice were anesthetized with 1% pentobarbital sodium (45 mg/kg, intraperitoneal) and subsequently euthanized by cervical dislocation. The tumor weight was measured and the volume was calculated at the ending point.

#### Autophagy flux detection

Harvest the exponential growth phase cells of CGA-T1-OE, shL3MBTL2 and corresponding control cells. Mix 2×10^5^ cells with 1 μg ptfLC3 plasmid (mRFP-GFP-LC3 plasmid, Addgene, USA) and applied to the routine nucleofection process. After the program is finished, cells were transferred to the 6-well plate with 1.5 mL culture medium for 36 h cell culture. Afterwards, cells were starved with EBSS medium (Gibco:14155063, USA) for 3 h and then the GFP and mRFP fluorescence in cells were observed using a confocal microscope (Leica DMI6000B-TCS-SP5 confocal system, USA). The numbers of red puncta (RFP^+^GFP^−^) versus yellow puncta (RFP^+^GFP^+^) per cell in each cell line were quantified. At least 30 cells in triplicate per condition were counted.

### Quantification and statistical analysis

The data are presented as the means ± SD from at least three independent experiments Statistical analysis was performed using GraphPad Prism 5.0 software (GraphPad Software, Inc., La Jolla, CA, USA). A two-tailed paired Student’s *t* test was used to assess the significance between two groups. Multiple groups were compared by a one-way or two-way ANOVA followed by Tukey's post-hoc test. Statistical significance was regarded as ∗p < 0.05 or ∗∗p < 0.01, ∗∗∗p < 0.001.

## Data Availability

The RNA-seq data generated during this study are available at Gene Expression Omnibus (GEO): GSE184834. This paper does not report original code. Any additional information required to reanalyze the data reported in this paper is available from the lead contact upon request.
