# Supplementary material for: L3MBTL2-mediated CGA transcriptional suppression promotes pancreatic cancer progression through modulating autophagy
Source: iScience. 2022 Apr 13;25(5):104249. doi: 10.1016/j.isci.2022.104249 (PMC9061862; doi:10.1016/j.isci.2022.104249)
Supplement: Document S1. Figures S1–S11 and Table S1 [file mmc1.pdf]

iScience, Volume *25*

Supplemental information

L3MBTL2-mediated CGA transcriptional suppression promotes pancreatic cancer progression through modulating autophagy

Hua Huang, Ruining Pan, Yue Zhao, Huan Li, Huiyu Zhu, Sijia Wang, Aamir Ali Khan, Juan Wang, and Xinhui Liu

Supplementary information

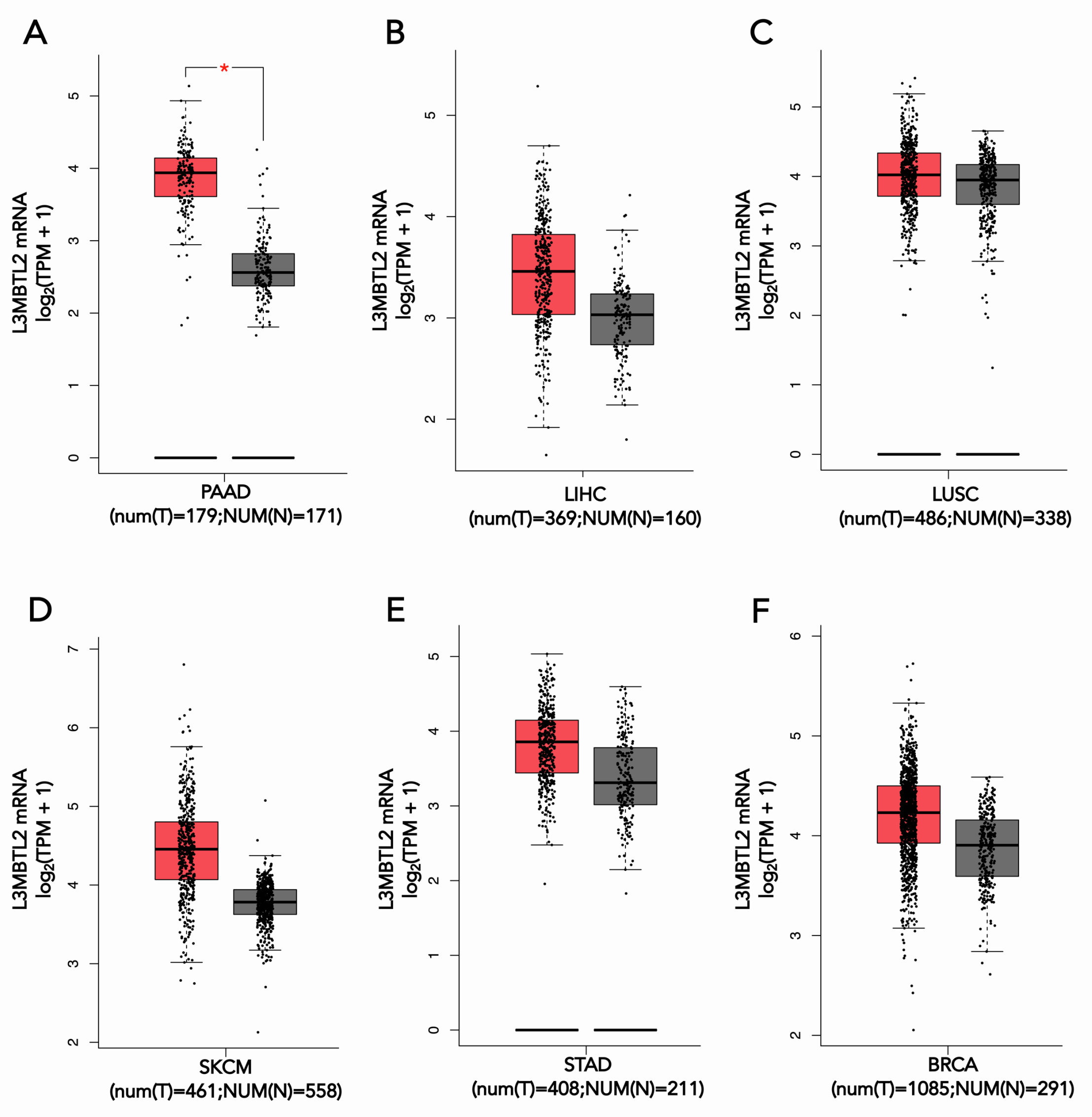

Figure S1. L3MBTL2 mRNA levels in different cancer tissues, Related to Figure 1.

A to **F,** Box plot showing the relative mRNA levels of L3MBTL2 in pancreatic cancer tissues (A), liver cancer tissues (B), lung cancer tissues (C), melanoma cancer tissues (D), gastric cancer tissues (E), breast cancer tissues (F) and their corresponding nontumor tissues according to the TCGA database.

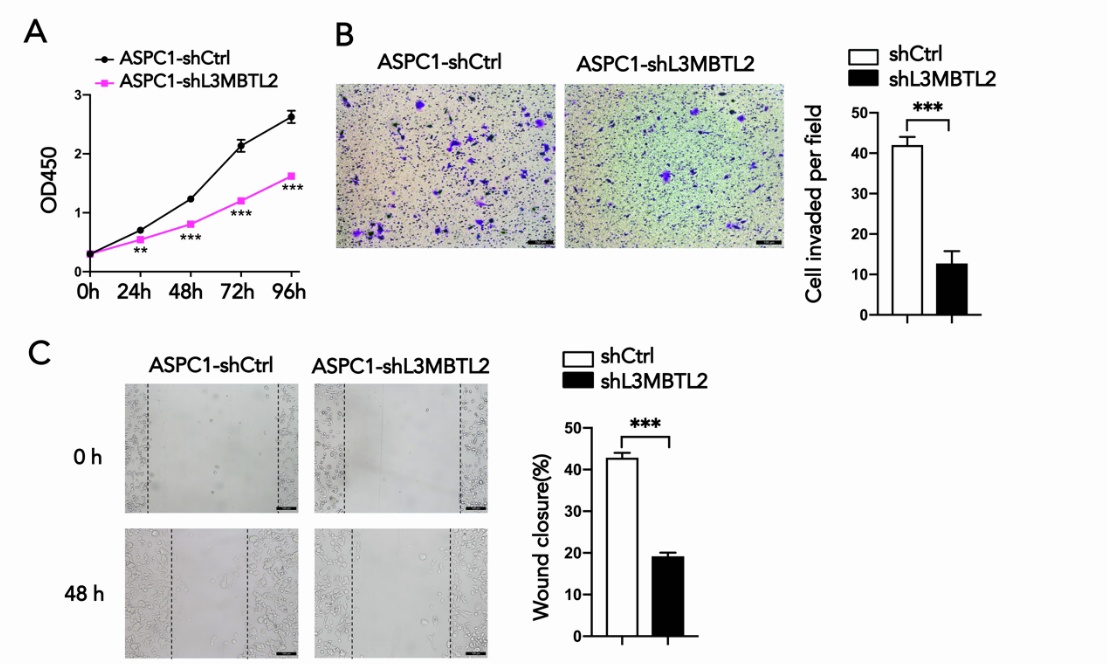

Figure S2. Knockdown L3MBTL2 inhibits the growth, migration and invasion of pancreatic cancer, Related to Figure 1.

A, Proliferation of ASPC-1 cells expressing shL3MBTL2 and shCtrl. **B and C,** Wound healing assays (C) and Transwell assays (B) of ASPC-1 cells expressing shL3MBTL2 and shCtrl. Scale bars, 100μm (B and C). Data are presented as means ± SD (n=3). *P <0.05, **P <0.01, *** P < 0.001, t-tests.

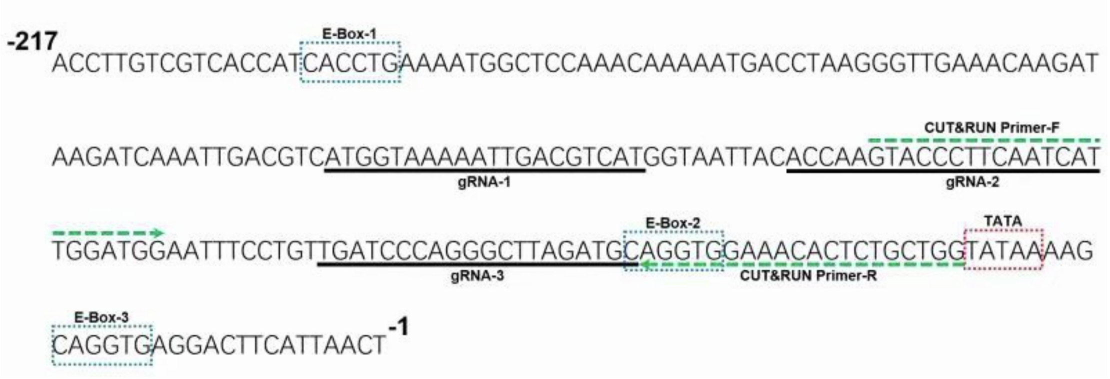

Figure S3. A sequence diagram showed the location of three E-Boxes, the primers for ChIP (cut & run) analysis and the guideRNAs' loci within the CGA promoter, Related to Figure 3 and Figure 4.

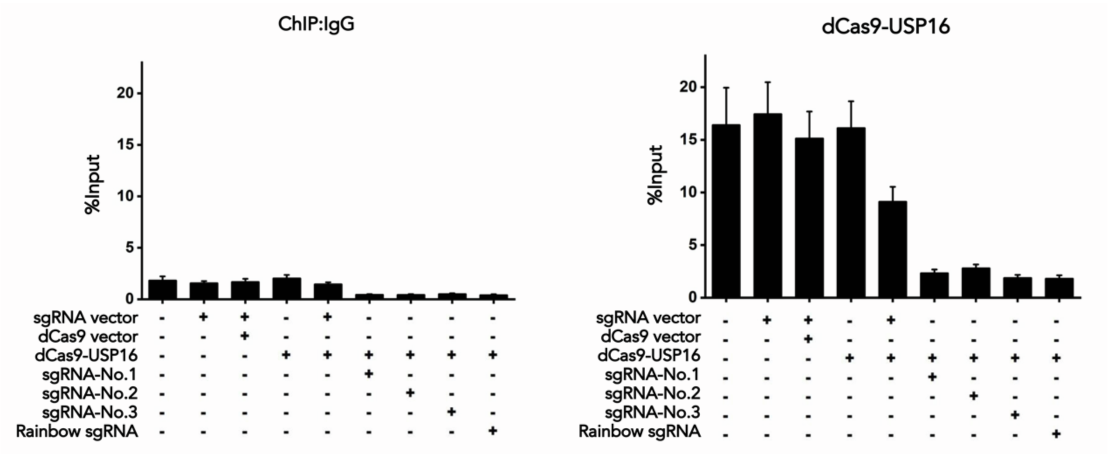

Figure S4. Cells transfected with dCas9-USP16 construct decreased the H2AK119ub1 at CGA promoter in the presence of gRNAs, Related to Figure 3.

Analysis of the H2AK119ub1 levels at the CGA promoter by ChIP-qPCR in 293T$^{RING1B\text{-}OE}$ cells co-transfected with either dCas9 fusion proteins (dCas9-USP16) or dCas9 empty vector and the indicated gRNAs. Data are presented as means ± SD (n=3). *P <0.05, **P <0.01, *** P < 0.001, t-tests.

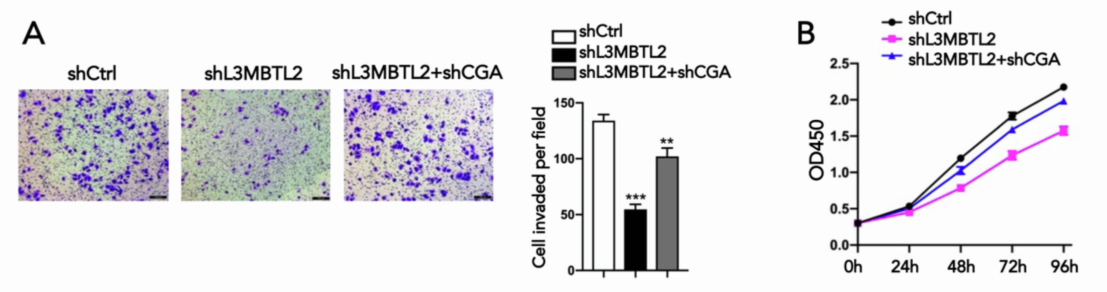

Figure S5. Functional correlation between L3MBTL2 and CGA in pancreatic cancer, Related to Figure 5.

A and **B,** The loss of CGA (shCGA) rescued the inhibition of cell proliferation (B) and invasion (A) caused by L3MBTL2 knockdown in PANC-1 cells. Scale bars, 100μm. Data are shown as the mean ± SD. n = 3 *P < 0.05, **P < 0.01 and *** P < 0.001 (t-tests) were considered significant.

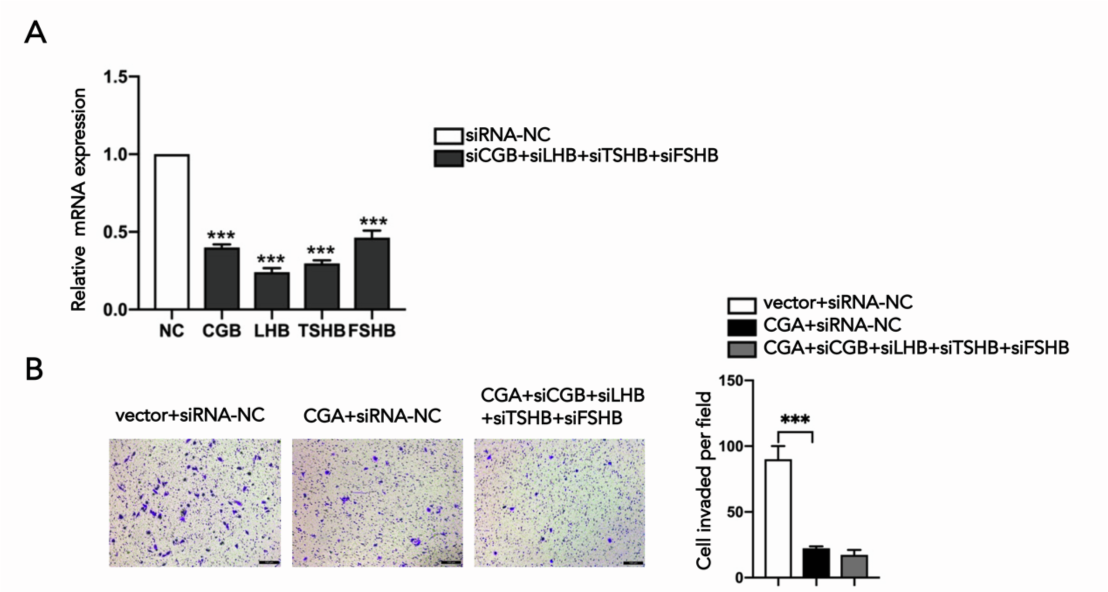

Figure S6. Downregulation of CGA corresponding beta subunits do not affect the anti-tumour effect of CGA overexpression in pancreatic cancer, Related to Figure 5.

A, The relative mRNA level of CGB, LHB, TSHB and FSHB in PANC-1 cells transfected with siRNA-NC and co-transfected with four siRNAs (siCGB, siLHB, siTSHB and siFSHB). **B,** The inhibition of CGB, LHB, TSHB and FSHB barely affected the inhibition of cell invasion caused by CGA overexpression in PANC-1 cells, scale bars, 100μm. Data are shown as the mean ± SD. n = 3 *P < 0.05, **P < 0.01 and *** P < 0.001 (t-tests) were considered significant.

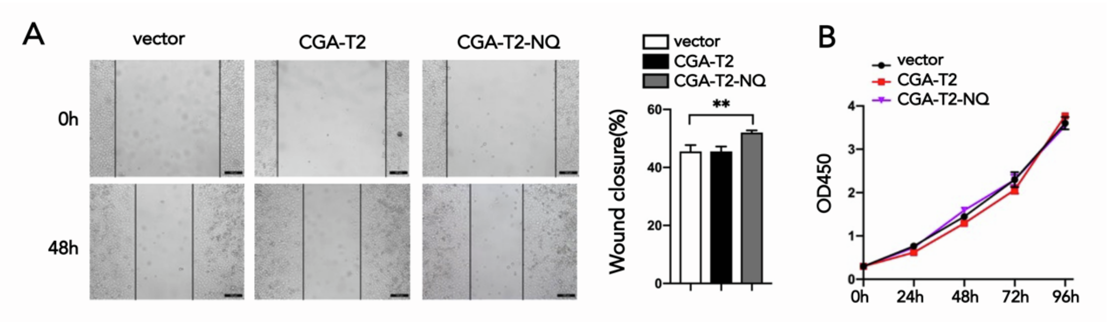

Figure S7. CGA-T2 presented no function on PDAC, Related to Figure 6.

A and **B,** wound healing (A) and proliferation (B) assays of PANC-1 cells expressing exogenous vector, CGA-T2 and CGA-T2-NQ. Scale bars, 100μm. Data are presented as means ± SD (n=3). *P <0.05, **P <0.01, *** P < 0.001, t-tests.

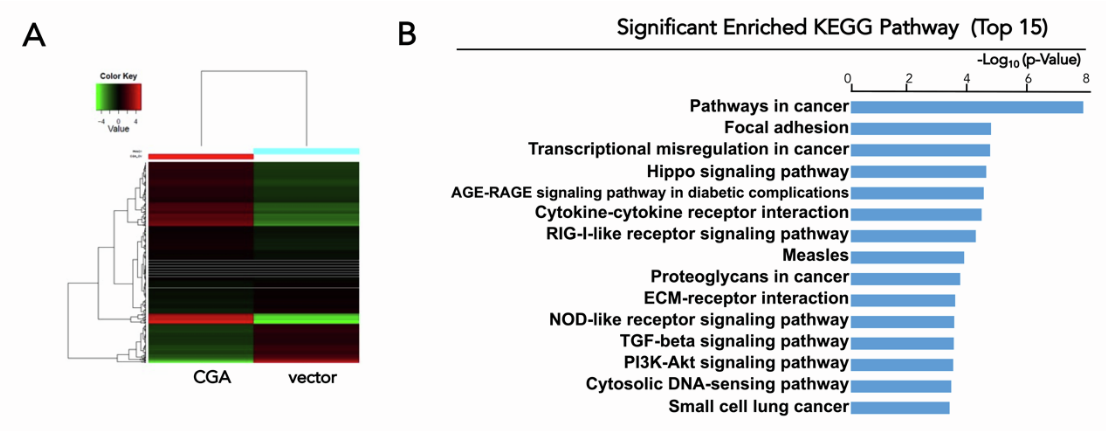

Figure S8. CGA involved in downstream pathways, Related to Figure 7.

A, RNA-seq was performed to identify the CGA downstream pathways using PANC-1 cells expressing exogenous CGA and vector **B,** The top 15 enriched KEGG pathways are shown and are ranked on the basis of the log10(P-value), according to RNA-seq results.

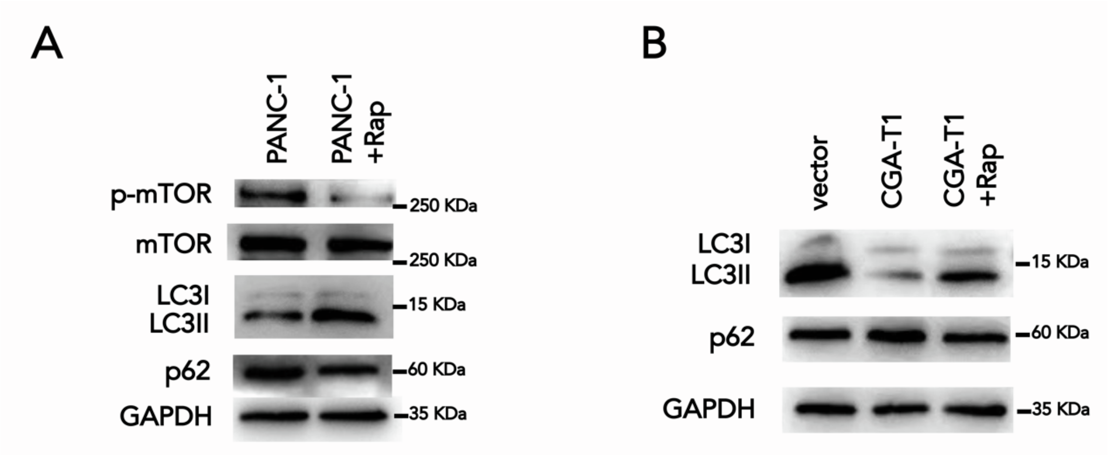

Figure S9. Glycosylated CGA-T1 inhibit the autophagy partially through mTOR pathway, Related to Figure 7.

A, mTOR inhibitor rapamycin increased LC3II and decreased P62 expression in PANC-1 cells compared to that of the control. **B**, Comparison of LC3II and P62 expression in PANC-1 cells transfected with vector or CGA-T1-OE, treated with or without rapamycin.

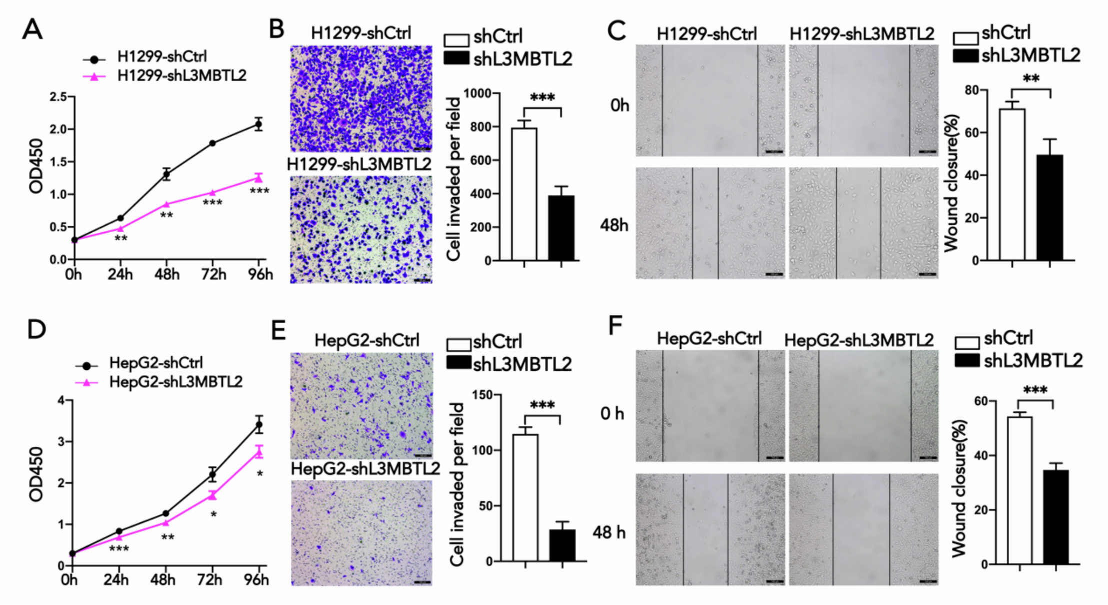

Figure S10. L3MBTL2 promotes tumor progression in lung and hepatology carcinoma, Related to Figure 1.

A to C, Cell proliferation (A), Transwell assays (B) and wound healing assays (C) of L3MBTL2-overexpressing H1299 cells. **D to F,** Cell proliferation (D), Transwell assays (E) and wound healing assays (F) of L3MBTL2-overexpressing HepG2 cells. Data are presented as means ± SD (n=3). *P <0.05, **P <0.01, *** P < 0.001, t-tests. Scale bars, 100μm (B, C, E and F).

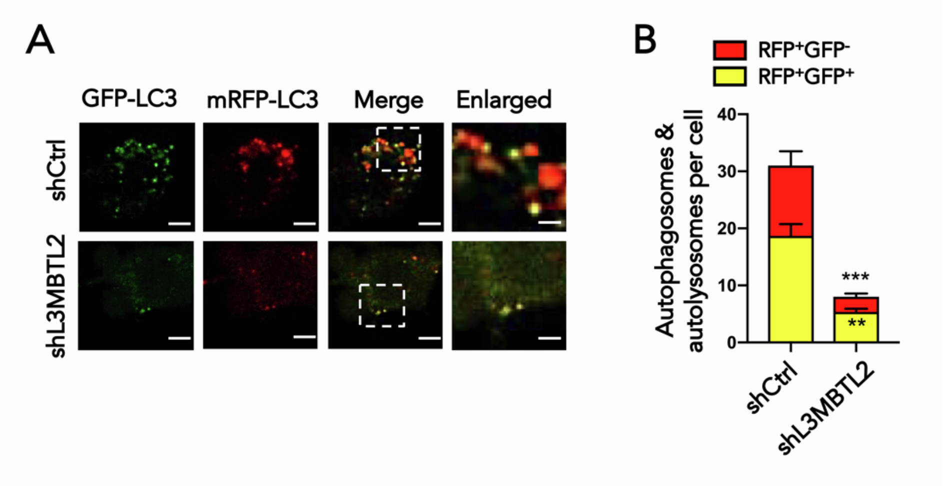

Figure S11. L3MBTL2 knockdown inhibits autophagic flux, Related to Figure 7.

A, The autophagic flux were detected in PANC-1-shCtrl and PANC-1-shL3MBTL2 cells that were transfected with mRFP-GFP-LC3 plasmid. Scale bar,5 μm, scale bar in enlarged image, 2 μm. **B,** The numbers of red puncta (RFP+GFP-) versus yellow puncta (RFP+GFP+) per cell in each cell line were quantified. Data are presented as means ± SD (n=3). *P <0.05, **P <0.01, *** P < 0.001, t-tests. Scale bars, 100μm.

Table S1. Primer sequences for quantitative PCR, Related to Figure 1 to Figure 4.

Target Gene	Primers (5'-3')
β-Actin	F: CATGTACGTTGCTATCCAGGC
	R: CTCCTTAATGTCACGCACGAT
L3MBTL2	F: TGAGAGCAGCTCCTATCTGGA
	R: TGTACCCACGATACCACACATC
CGA	F: CACTCCACTAAGGTCCAAGAAGA
	R: CCGTGTGGTTCTCCACTTTGA
CGA promoter	F: GTACCCTTCAATCATTGGATGG
	R: CCAGCAGAGTGTTTCCACCT

F, Forward; R, Reverse
